# Hypofractionated radiotherapy versus conventionally fractionated radiotherapy for patients with intermediate-risk localised prostate cancer: 2-year patient-reported outcomes of the randomised, non-inferiority, phase 3 CHHiP trial

**DOI:** 10.1016/S1470-2045(15)00280-6

**Published:** 2015-12

**Authors:** Anna Wilkins, Helen Mossop, Isabel Syndikus, Vincent Khoo, David Bloomfield, Chris Parker, John Logue, Christopher Scrase, Helen Patterson, Alison Birtle, John Staffurth, Zafar Malik, Miguel Panades, Chinnamani Eswar, John Graham, Martin Russell, Peter Kirkbride, Joe M O'Sullivan, Annie Gao, Clare Cruickshank, Clare Griffin, David Dearnaley, Emma Hall

**Affiliations:** a The Institute of Cancer Research, London, UK; b Clatterbridge Cancer Centre, Wirral, UK; c Royal Marsden NHS Foundation Trust, London, UK; d Brighton and Sussex University Hospitals, Brighton, UK; e Christie Hospital, Manchester, UK; f Ipswich Hospital, Ipswich, UK; g Addenbrooke's Hospital, Cambridge, UK; h Rosemere Cancer Centre, Royal Preston Hospital, UK; i Cardiff University, Cardiff, UK; j Velindre Cancer Centre, Cardiff, UK; k Whiston Hospital, Prescot, UK; l Lincoln County Hospital, Lincoln, UK; m Southport General Infirmary, Southport, UK; n Beacon Centre, Musgrove Park Hospital, Taunton, UK; o Beatson West of Scotland Cancer Centre, Glasgow, UK; p Sheffield Teaching Hospitals Foundation Trust, Sheffield, UK; q Queen's University Belfast, Belfast, UK

## Abstract

**Background:**

Patient-reported outcomes (PROs) might detect more toxic effects of radiotherapy than do clinician-reported outcomes. We did a quality of life (QoL) substudy to assess PROs up to 24 months after conventionally fractionated or hypofractionated radiotherapy in the Conventional or Hypofractionated High Dose Intensity Modulated Radiotherapy in Prostate Cancer (CHHiP) trial.

**Methods:**

The CHHiP trial is a randomised, non-inferiority phase 3 trial done in 71 centres, of which 57 UK hospitals took part in the QoL substudy. Men with localised prostate cancer who were undergoing radiotherapy were eligible for trial entry if they had histologically confirmed T1b–T3aN0M0 prostate cancer, an estimated risk of seminal vesicle involvement less than 30%, prostate-specific antigen concentration less than 30 ng/mL, and a WHO performance status of 0 or 1. Participants were randomly assigned (1:1:1) to receive a standard fractionation schedule of 74 Gy in 37 fractions or one of two hypofractionated schedules: 60 Gy in 20 fractions or 57 Gy in 19 fractions. Randomisation was done with computer-generated permuted block sizes of six and nine, stratified by centre and National Comprehensive Cancer Network (NCCN) risk group. Treatment allocation was not masked. UCLA Prostate Cancer Index (UCLA-PCI), including Short Form (SF)-36 and Functional Assessment of Cancer Therapy-Prostate (FACT-P), or Expanded Prostate Cancer Index Composite (EPIC) and SF-12 quality-of-life questionnaires were completed at baseline, pre-radiotherapy, 10 weeks post-radiotherapy, and 6, 12, 18, and 24 months post-radiotherapy. The CHHiP trial completed accrual on June 16, 2011, and the QoL substudy was closed to further recruitment on Nov 1, 2009. Analysis was on an intention-to-treat basis. The primary endpoint of the QoL substudy was overall bowel bother and comparisons between fractionation groups were done at 24 months post-radiotherapy. The CHHiP trial is registered with ISRCTN registry, number ISRCTN97182923.

**Findings:**

2100 participants in the CHHiP trial consented to be included in the QoL substudy: 696 assigned to the 74 Gy schedule, 698 assigned to the 60 Gy schedule, and 706 assigned to the 57 Gy schedule. Of these individuals, 1659 (79%) provided data pre-radiotherapy and 1444 (69%) provided data at 24 months after radiotherapy. Median follow-up was 50·0 months (IQR 38·4–64·2) on April 9, 2014, which was the most recent follow-up measurement of all data collected before the QoL data were analysed in September, 2014. Comparison of 74 Gy in 37 fractions, 60 Gy in 20 fractions, and 57 Gy in 19 fractions groups at 2 years showed no overall bowel bother in 269 (66%), 266 (65%), and 282 (65%) men; very small bother in 92 (22%), 91 (22%), and 93 (21%) men; small bother in 26 (6%), 28 (7%), and 38 (9%) men; moderate bother in 19 (5%), 23 (6%), and 21 (5%) men, and severe bother in four (<1%), three (<1%) and three (<1%) men respectively (74 Gy *vs* 60 Gy, p_trend_=0.64, 74 Gy *vs* 57 Gy, p_trend_=0·59). We saw no differences between treatment groups in change of bowel bother score from baseline or pre-radiotherapy to 24 months.

**Interpretation:**

The incidence of patient-reported bowel symptoms was low and similar between patients in the 74 Gy control group and the hypofractionated groups up to 24 months after radiotherapy. If efficacy outcomes from CHHiP show non-inferiority for hypofractionated treatments, these findings will add to the growing evidence for moderately hypofractionated radiotherapy schedules becoming the standard treatment for localised prostate cancer.

**Funding:**

Cancer Research UK, Department of Health, and the National Institute for Health Research Cancer Research Network.

**Research in context**

**Evidence before this study**

Randomised controlled trials of moderately hypofractionated radiotherapy schedules versus conventionally fractionated radiotherapy for localised prostate cancer, using both older and more modern radiotherapy techniques, have shown inconsistent results for both efficacy and side-effects. These studies have not usually included health-related quality of life or patient-reported outcomes (PROs), which detect more side-effects than do clinician-reported outcomes. We searched PubMed using the terms “patient-reported outcomes” OR “quality of life” AND “hypofractionated” OR “hypofractionation” AND “prostate” up to Oct 1, 2002, and retrieved eight articles. Of these articles, none reported PROs from randomised trials of conventional versus hypofractionated radiotherapy.

**Added value of this study**

To our knowledge, this study is the largest randomised trial of moderately hypofractionated versus conventionally fractionated radiotherapy using modern radiotherapy techniques, and the first to report PROs up to 2 years after treatment, showing both early and late developing side-effects. Other randomised trials that have included PROs and have been reported since this study began used older radiotherapy techniques, or only included follow-up to 3 months after radiotherapy, therefore assessing early rather than late treatment effects, which are usually dose-limiting.

**Implications of all the available evidence**

If efficacy outcomes from CHHiP show non-inferiority for hypofractionated treatments, the absence of any difference in PROs between trial groups adds to the growing evidence for moderately hypofractionated radiotherapy schedules becoming the standard treatment for localised prostate cancer.

## Introduction

Prostate cancer is the most common cancer in men in the UK, with 41 700 patients diagnosed in 2011.^1^ For patients diagnosed with localised disease, external beam radiotherapy, radical prostatectomy, and brachytherapy are conventional treatments with similar control rates for organ-confined tumours. Management choices are therefore often affected by potential treatment-related toxic effects. Patient-reported outcomes (PROs) detect treatment side-effects more reliably than do clinician-reported measures and might better guide treatment decisions.^2^, ^3^

The Conventional or Hypofractionated High Dose Intensity Modulated Radiotherapy in Prostate Cancer (CHHiP) trial (CRUK/06/016) randomly assigned men with localised prostate cancer who were undergoing radiotherapy to a standard fractionation schedule or to one of two hypofractionated regimens. The main aims of the trial were to compare the efficacy and toxic effects of conventional and hypofractionated radiotherapy. Quality of life (QoL) was assessed in a substudy within the main trial, in which we aimed to assess whether PROs differed between patients receiving conventionally fractionated versus hypofractionated radiotherapy up to 24 months after radiotherapy.

## Methods

### Study design and participants

CHHiP was a randomised, non-inferiority phase 3 trial done in three seamless stages. Participation in the QoL substudy was open to all UK centres participating in the main trial. Because it recruited ahead of schedule, the QoL substudy closed to accrual before the main trial closed.

Men older than 16 years who had histologically confirmed T1b–T3aN0M0 prostate cancer and a WHO performance status 0 or 1 were eligible for participation. Initially, men with a prostate-specific antigen (PSA) concentration of less than 40 ng/mL and risk of lymph node involvement less than 30% were eligible; on Aug 1, 2006, these criteria were revised and a PSA concentration less than 30 ng/mL and a risk of seminal vesicle involvement less than 30% were needed. Patients were ineligible if they had T3 tumours and a Gleason score of 8 or higher, or a life expectancy of less than 10 years. Full details of trial design, eligibility, and treatment have been reported previously.^4^

The study was approved by the London Multi-centre Research Ethics Committee (04/MRE02/10). It was sponsored by the Institute of Cancer Research and was done in accordance with the principles of good clinical practice. All patients provided written informed consent. The Institute of Cancer Research Clinical Trials and Statistics Unit (ICR-CTSU; Sutton, UK) coordinated the study and carried out central statistical data monitoring and all analyses. The trial management group was overseen by an independent trial steering committee.

### Randomisation and masking

Men were registered in the trial before or after starting initial hormone therapy. 4–6 weeks before radiotherapy, participants were randomly assigned (1:1:1) to receive a standard fractionation (control) or one of two hypofractionated schedules. Randomisation was done centrally via telephone calls to the ICR-CTSU. Computer-generated random permuted blocks were used, with block sizes of six and nine. Patients were stratified by centre and National Comprehensive Cancer Network (NCCN) risk group. Neither treatment allocation nor clinical assessment were masked because sham radiotherapy was not given.

### Procedures

Men with NCCN intermediate-risk or high-risk disease received short-course androgen suppression for 3–6 months before and during radiotherapy; this was optional for patients with low-risk disease. Individuals assigned to the control group received standard radiotherapy with 2 Gy daily fractions (Monday to Friday treatment) for 7·4 weeks, to give a total dose of 74 Gy in 37 fractions. Individuals in the experimental groups received hypofractionated treatment with 3 Gy daily fractions to a total dose of either 60 Gy in 20 fractions in 4·0 weeks or 57 Gy in 19 fractions in 3·8 weeks. For the hypofractionated schedules, the protocol stated that the overall duration of treatment should be at least 28 days for the 20-fraction schedule and at least 27 days for the 19-fraction schedule. This was to avoid undue shortening of overall treatment time and, in practice, meant that treatment started on a Wednesday to Friday. Forward or inverse three-dimensional methods were used to plan radiotherapy treatment. Further details of treatment and quality assurance have been reported previously.^4^

Men consenting to participate in the QoL substudy were eligible to complete questionnaires at trial entry if they had not already started endocrine treatment, to minimise the effect of toxicity of hormone deprivation on QoL at this timepoint. All men were eligible to complete further questionnaires pre-radiotherapy, and at 10 weeks and 6, 12, 18, and 24 months after the start of radiotherapy. From trial entry to 6 months after radiotherapy, questionnaires were administered in the clinic, and subsequent questionnaires were posted to patients from the ICR-CTSU after local verification of their current health status. All QoL questionnaires were self-administered.

During the planning stages of the CHHiP trial, the University of California, Los Angeles Prostate Cancer Index (UCLA-PCI) was an important QoL instrument available for use in patients with localised prostate cancer.^5^ Subsequently it became apparent that this instrument needed augmentation to better capture the broad range of urinary, bowel, sexual, and hormonal symptoms in patients receiving external beam radiotherapy or brachytherapy, or undergoing radical prostatectomy. Consequently the Expanded Prostate Cancer Index Composite (EPIC) QoL instrument was developed that had item content that better represented typical symptoms after radiotherapy.^6^ To maximise the sensitivity of the PROs, the QoL instruments were updated during the trial to include the EPIC instrument. Therefore from trial initiation to early 2009, the UCLA-PCI, including the Short Form 36 (SF-36) and Functional Assessment of Cancer Therapy-Prostate (FACT-P) QoL instruments were used.^7^ Following a protocol amendment on March 12, 2009, the EPIC and Short Form 12 (SF-12) QoL instruments replaced UCLA-PCI, SF-36, and FACT-P, although some old questionnaires were received back from participants after this date.^8^ EPIC-50 was used for bowel and urinary domains and EPIC-26 for sexual and hormonal domains.^9^

UCLA-PCI consists of 20 items organised into six domains, including bowel function (four items), bowel bother (one item), urinary function (five items), urinary bother (one item), sexual function (eight items), and sexual bother (one item). EPIC-50 includes a bowel function domain (seven items) and a bowel bother domain (seven items), which together form the bowel summary domain, and a urinary function domain (five items) and a urinary bother domain (seven items), which together form a urinary summary domain. EPIC-26 includes a sexual function domain (five items) and a sexual bother domain (one item), which are combined to form a sexual summary domain; there is also a hormonal domain (five items). Items in the UCLA-PCI and EPIC QoL instruments differed: for example, the EPIC bowel function domain included rectal bleeding, faecal incontinence, and daily bowel movements, which were absent from UCLA-PCI, whereas bowel distress was represented in the UCLA-PCI bowel function domain and absent from EPIC-50. Additionally, haematuria and dysuria were represented in the EPIC urinary function domain, but absent from UCLA-PCI. All QoL instrument scores range from 0 to 100 and a higher score represents better QoL.

Health-related QoL was assessed using the FACT-P and SF-36 instruments (with UCLA-PCI) or the SF-12 instrument (with EPIC). FACT-P consists of physical, social, functional, and emotional wellbeing domains; each domain has seven items, and scores per domain range from 0 to 28, except for emotional wellbeing, which ranges from 0 to 24. The SF-36 instrument includes eight domains of physical functioning, social functioning, vitality, role limitations (physical), role limitations (emotional), mental health, general health, and bodily pain, and each domain score ranges from 0 to 100. SF-12 consists of a physical composite score (PCS) and mental composite score (MCS), each of which have six items, and scores range from 0 to 100. For all three instruments, a higher score represents better quality of life. All questionnaires were scored in accordance with the recommended scoring manuals.^10^, ^11^, ^12^, ^13^, ^14^ Not all respondents answered all questions; all available data points were used in analyses.

Separate QoL analyses were planned after 2 and 5 years of follow-up, and this report describes PROs up to 2 years.

### Outcomes

The primary endpoint was the single item “Overall how much of a problem have your bowels been for you during the last 4 weeks” (overall bowel bother). This question was chosen because it gives a good overall measure of bowel-associated morbidity and is common to both the UCLA-PCI and EPIC instruments, so was reported by all patients. The main secondary endpoints were overall urinary bother and overall sexual bother. Additional secondary endpoints were individual bowel, urinary, and sexual items and domain scores assessed within EPIC and UCLA-PCI and the general health-related QoL domain scores in FACT-P, SF-36, and SF-12.

### Statistical analysis

For all endpoints, the control group was compared with each of the experimental groups, as per the statistical analysis plan of the main trial. Formal statistical tests were done at 24 months. After this analysis, a post-hoc pragmatic comparison was done between the 60 Gy and 57 Gy experimental schedules to support clinical management choices if both hypofractionated schedules were shown to be non-inferior to standard fractionation for disease control. With 443 patients per experimental group, this substudy would have 80% power and 2·5% two-sided significance to detect changes in the proportion of patients with overall bowel bother scores as follows: from 65% in the standard fractionation group to 60% in an experimental hypofractionation group for scores of 1 (no bother), from 22% to 20% for scores of 2 (very small bother), from 7% to 10% for scores of 3 (small bother), and from 6% to 10% for scores of 4 or 5 (moderate or severe bother). Because the trial was not originally powered for QoL analyses, these calculations were done retrospectively, but before any analysis occurred. Power calculations were based on comparisons of two independent groups of ordered categorical data, with constant odds ratios computed across all categories, and assumed complete data would be available for 70% of patients (1330 individuals) at 2 years. A significance level of 0·001 and 99% confidence intervals were used, guided by a Bonferroni adjustment, to make some allowance for multiple testing.

We did cross-sectional, time-to-event, and change-from-baseline analyses. Cross-sectional analysis was done at each timepoint, with formal comparisons between treatment groups at 24 months via the χ^2^ test for trend and the Mann-Whitney *U* test. We combined moderate and severe events for the formal comparisons because of the small number of severe events. We did time-to-event analysis using Kaplan-Meier methods and the log-rank test to assess time to small or worse, and moderate or worse events for individual items. This analysis aimed to detect differences in late radiation toxic effects between treatment groups and therefore did not include the 10-week post-radiotherapy assessment, which assessed acute symptoms. Time-to-event was therefore measured from the start of radiotherapy to the QoL assessments at 6, 12, 18, or 24 months. Patients who reached the relevant endpoint at trial entry or pre-radiotherapy were excluded from that specific time-to-event analysis.

We assessed change from baseline (post-radiotherapy score minus baseline score) to account for differences in pre-existing comorbidity between groups. For bowel and urinary items and domain scores, we used the pre-radiotherapy score as a surrogate baseline score unless it was missing, in which case the baseline score was used. We used this surrogate to maximise numbers. To assess the sensitivity of the results to this assumption, analyses were repeated using baseline data only. For sexual endpoints, only the baseline score at trial entry was used. A sensitivity analysis was also done to confirm the robustness of including the baseline assessments of patients receiving less than 1 month of endocrine treatment which involved repeating analyses using baseline assessments of patients receiving no endocrine therapy beforehand.

**Figure 1:**
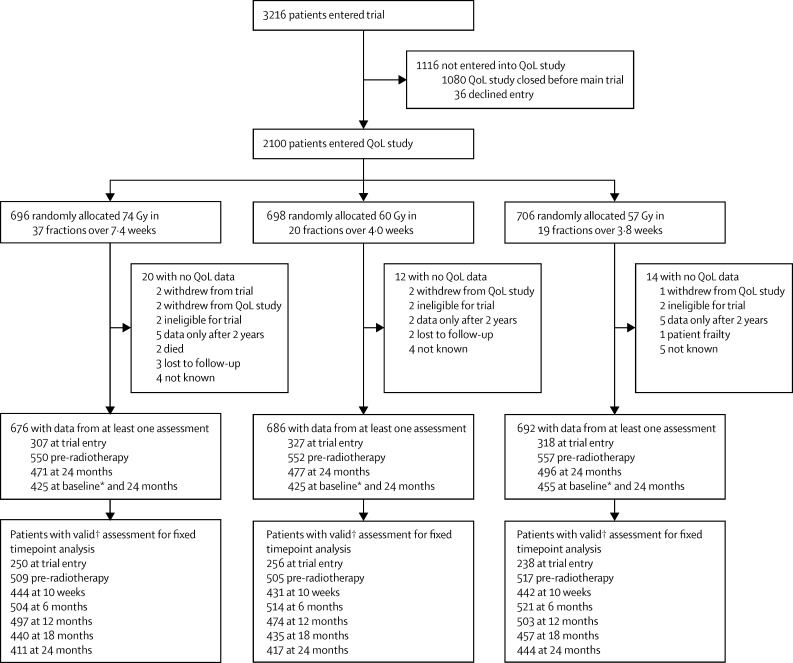
Quality-of-life study profile *Trial entry or pre-radiotherapy if patients were receiving endocrine therapy at trial entry. †Patients were excluded from the fixed timepoint analyses if their QoL assessments were dated outside prespecified acceptable time intervals: after 1 month of endocrine treatment or after randomisation for baseline; before 3 months or after 1 week of starting radiotherapy for pre-radiotherapy; outside 2 weeks from the expected date of completion for 10 weeks; and outside of 3 months from the expected date of completion for later timepoints. QoL=quality of life.

We modelled the odds of any specific change from baseline or pre-radiotherapy to 24 months using ordinal logistic regression after checking the validity of the proportional odds assumption.^15^ Odds ratios less than one favour the relevant experimental group. For the ordinal logistic regression models, the dependent variable is the post-radiotherapy score minus the baseline or pre-radiotherapy score, taking values of −4, −3, −2, −1, 0, 1, 2, 3, or 4, where negative numbers represent an improvement in QoL and positive numbers represent worsening QoL. We used ANCOVA modelling to assess change from baseline for continuous variables such as domain scores, adjusting for baseline or pre-radiotherapy score as indicated above. We assessed the normality assumption of the ANCOVA model visually via histograms and we did not deem formal tests to be necessary. Patients were excluded from the fixed timepoint analyses if their QoL assessments were dated outside prespecified acceptable time intervals, as outlined in figure 1.

No imputation of missing PRO data was done. For missing individual items, domain scores were only calculated if sufficient items were completed in accordance with the relevant scoring manual. For absent whole instruments, the effect of these missing data was assessed by comparison of the baseline characteristics of patients present in the analysis versus, first, those who consented but were missing entirely, and second, those who consented but were missing at 24 months, when formal statistical testing was done.

Analysis was on an intention-to-treat basis and all analyses were done with Stata version 13.1. The CHHiP trial is registered as an International Standard Randomised Controlled Trial, number ISRCTN97182923.

### Role of the funding source

The funders provided peer-reviewed approval for the study concept but had no role in study design, data collection, data analysis, data interpretation, or writing of the report. AW, HM, CG, and EH had access to all the raw data. The lead and corresponding authors had full access to all data in the study and had final responsibility for the decision to submit for publication.

## Results

Between Oct 18, 2002, and Nov 1, 2009, 2100 patients were recruited from 57 centres in the UK (figure 1 and appendix p 14) into the QoL substudy of the CHHiP trial; subsequently, the substudy closed to accrual. 696 patients were assigned to the standard 74 Gy schedule, 698 were assigned to the 60 Gy schedule, and 706 were assigned to the 57 Gy schedule.

Median follow-up was 50·0 months (IQR 38·4–64·2) on April 9, 2014, which was the most recent follow-up measurement of all data collected before the QoL data snapshot for this analysis in September, 2014. At trial entry, 700 (86%) of the 812 eligible patients who had not started endocrine treatment plus 252 patients who had started endocrine treatment returned questionnaires. Questionnaires were returned by 1659 (79%) patients pre-radiotherapy, 1470 (70%) patients at 10 weeks, 1597 (76%) patients at 6 months, 1551 (74%) patients at 12 months, 1456 (69%) patients at 18 months, and 1444 (69%) patients at 24 months.

Baseline characteristics of patients were balanced between treatment groups except for an imbalance in T stage between the 74 Gy and 60 Gy groups (table 1). 1490 (71%) patients had intermediate NCCN risk disease.^16^

For 46 patients reported to have consented to enter the substudy, no QoL assessments were received by the ICR-CTSU. Baseline characteristics for these patients were not significantly different from those of patients present in the analysis (appendix p 1). 828 (39%) patients who had consented to participate in the substudy had no QoL assessments available at 24 months. The only significant difference in baseline characteristics between groups with and without 24-month PRO data was that patients with missing questionnaires were more likely to have high NCCN risk disease at trial entry than were patients who provided data (appendix p 2). Of patients with data from at least one QoL assessment, 665 (98%) of 676 in the 74 Gy treatment group, 674 (98%) of 686 in the 60 Gy treatment group, and 677 (98%) of 692 in the 57 Gy treatment group received endocrine therapy.

**Figure 2:**
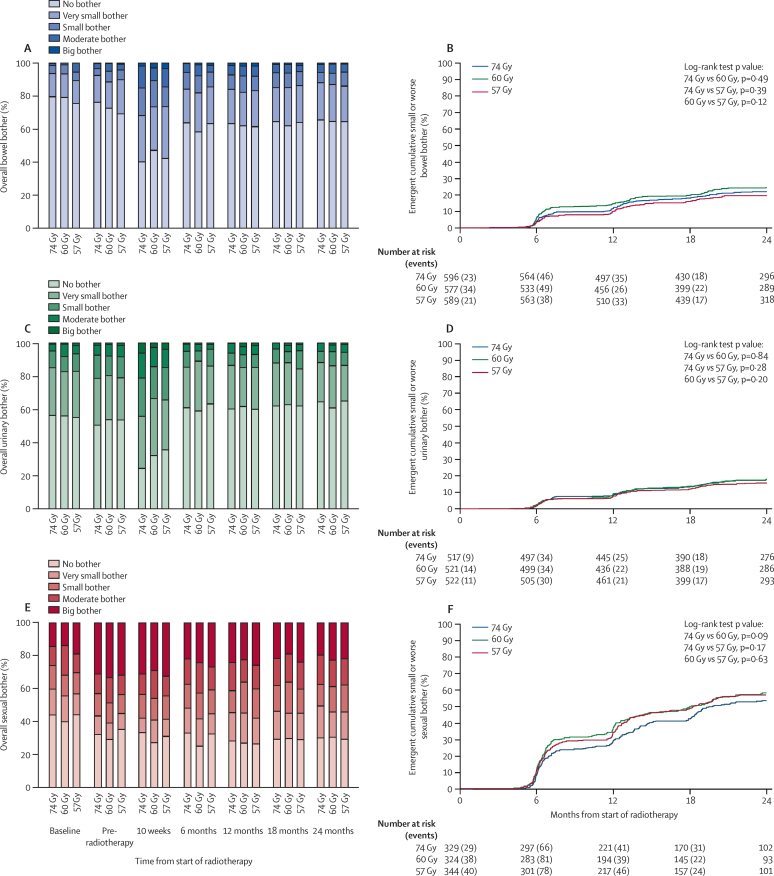
Overall bowel, urinary, and sexual bother Data are prevalence of overall bowel bother (A), time to small or worse overall bowel bother (B), prevalence of overall urinary bother (C), time to small or worse overall urinary bother (D), prevalence of overall sexual bother (E), and time to small or worse overall sexual bother (F).

The incidence of overall bowel bother was low (figure 2, appendix p 3). At 24 months post-radiotherapy, we recorded no overall bowel bother for 269 (66%) of 410 men treated with 74 Gy, 266 (65%) of 411 men treated with 60 Gy, and 282 (65%) of 437 men treated with 57 Gy; very small bother for 92 (22%), 91 (22%), and 93 (21%) men; small bother for 26 (6%), 28 (7%), and 38 (9%) men; moderate bother for 19 (5%), 23 (6%), 21 (5%) men; and severe bother for four (<1%), three (<1%), and three (<1%) men, respectively. Cross-sectional analysis at 24 months showed no significant differences in overall bowel bother between the treatment groups (74 Gy *vs* 60 Gy, p_trend_=0·64; 74 Gy *vs* 57 Gy, p_trend_=0·59; appendix p 3).

A temporary increase in any bowel bother was seen at 10 weeks (a change from 413 [27%] of 1509 patients pre-radiotherapy to 745 [57%] of 1309 patients at 10 weeks). At 6 months, any bowel bother had decreased (581 [38%] of 1519 patients), and remained around this level to 24 months, when any overall bowel bother was reported by 441 (35%) of 1258 patients. A sensitivity analysis showed that using pre-radiotherapy scores as a surrogate for baseline scores at trial entry for some patients was valid (appendix p 11–12). Because 252 men completed baseline questionnaires after starting endocrine treatment, a sensitivity analysis was done, which confirmed the robustness of including patients receiving less than 1 month of endocrine treatment at baseline (appendix p 13).

The pattern in overall urinary bother was similar to that for overall bowel bother (figure 2, appendix p 4) and cross-sectional analysis at 24 months showed no significant differences in overall urinary bother between treatment groups (appendix p 4). The baseline incidence of overall sexual bother was higher than that for bowel or urinary bother, with 412 (57%) of 719 patients having any bother at baseline, which increased to 975 (68%) of 1440 patients pre-radiotherapy and improved from 6 months to 24 months (figure 2, appendix p 4). There were no significant differences between treatment groups

At 24 months, we noted no significant differences between treatment groups for all other individual bowel, urinary, and sexual items assessed (appendix pp 3–4). Bowel, urinary, and sexual domain scores assessed within UCLA-PCI or EPIC QoL instruments also showed no difference between treatments at 24 months (table 2).

Time-to-event analysis of small or worse overall bowel, urinary, and sexual bother showed no significant differences between treatment groups for any endpoints (figure 2). The appendix contains absolute numbers of cumulative small or worse and moderate or worse events, the prevalence of the relevant bowel, urinary, and sexual symptoms before radiotherapy, and the hazard ratios for the time-to-event analysis time from start of radiotherapy to small or worse and moderate or worse events for all individual items in all treatment groups (appendix pp 5 and 6). The number of patients reporting symptoms that were represented only in EPIC (faecal incontinence, rectal bleeding, daily bowel movements, dysuria, and haematuria) was considerably lower than that for other endpoints, so these analyses were underpowered.

Although we saw no significant differences between treatment groups, the cumulative incidence of some symptoms, including faecal incontinence, rectal bleeding, and use of urinary pads, was higher in patients treated with hypofractionated radiation than in those treated with standard fractionation (appendix p 5). However, at 24 months, differences in the prevalence of these symptoms between groups were smaller (appendix pp 3–4).

**Figure 3:**
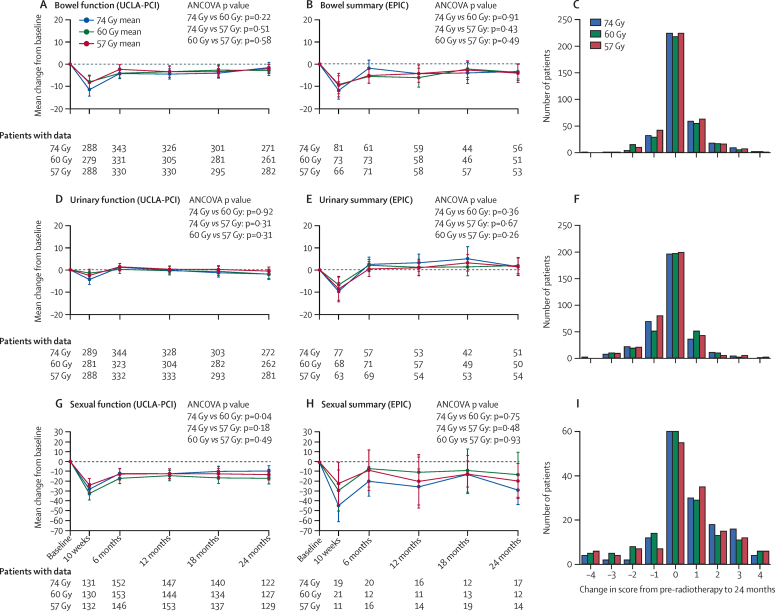
Change in domain scores and single item overall bother scores from baseline to 24 months Change in UCLA-PCI bowel function domain score*† (A); change in EPIC bowel summary domain score*† (B); change in overall bowel bother from pre-radiotherapy† to 24 months (C); change in UCLA-PCI urinary function domain score*† (D); change in EPIC urinary summary domain score*† (E); change in overall urinary bother from pre-radiotherapy† to 24 months (F); change in UCLA-PCI sexual function domain score* (G); change in EPIC sexual summary domain score* (H); and change in overall sexual bother from baseline to 24 months (I). EPIC=Expanded Prostate Cancer Index Composite. UCLA-PCI=University of California, Los Angeles Prostate Cancer Index. Error bars are 99% CIs.*Higher domain scores indicate better function. †For all urinary and bowel items and domain scores, to maximise numbers, the pre-radiotherapy score was used as a surrogate baseline score unless missing, in which case the baseline score was used.

Figure 3 shows change from baseline for UCLA-PCI domain scores and EPIC domain summary scores; additional EPIC domain scores are shown in the appendix (p 10). There were no significant differences between treatment groups. For all urinary and bowel items and domain scores, to maximise numbers, the pre-radiotherapy score was used as a surrogate baseline score unless missing, in which case the baseline score was used; exact numbers are: 749 pre-radiotherapy plus 65 baseline for change in UCLA-PCI bowel function to 24 months; 146 plus 14 for change in EPIC bowel summary to 24 months; 751 plus 64 for change in UCLA-PCI urinary function to 24 months; and 139 plus 16 for change in EPIC urinary summary to 24 months (numbers per treatment group shown in appendix p 14).

Figure 3 also shows change from baseline in scores for the individual items of overall bowel bother, overall urinary bother, and overall sexual bother; all other endpoints are shown in the appendix (p 8). Most patients had no change in score from baseline and we noted no significant differences between treatment groups in change from baseline to 24 months for any individual items. Compared to the 74 Gy control group, the odds of a one-point increase in overall bowel bother were reduced, although not significantly, in the 60 Gy treatment group (odds ratio [OR] 0·85 [99% CI 0·57–1·26]; p=0·29) and the 57 Gy treatment group (OR 0·84 [0·57–1·24]; p=0·25). For some endpoints, the odds of a patient developing side-effects were slightly increased for the 60 Gy group compared with the 57 Gy group, but none of these increases were significant (appendix p 8). Again for urinary and bowel overall bother items, the pre-radiotherapy score was used as a surrogate for baseline. Exact numbers are 971 pre-radiotherapy plus 85 baseline for overall bowel bother; 969 plus 84 for overall urinary bother (per treatment group shown in appendix p 14).

We identified no significant differences in health-related QoL domain scores measured by FACT-P, SF-12, and SF-36 between treatment groups at 24 months (appendix p 9). For most domains, there was a consistent pattern of stable scores across all timepoints, including pre-radiotherapy and 10 weeks post-radiotherapy timepoints. However, for the SF-36 domains of vitality, physical role functioning, and social wellbeing, we noted a reduction of more than 10 points between the median domain score at baseline and at 10 weeks after the start of radiotherapy in all treatment groups. By 6 months, median scores had increased to within 10 points of the median scores at baseline for all three measures.

## Discussion

In this QoL substudy of the CHHiP trial, PROs were not significantly different between treatment groups for any of the endpoints assessed. Both cross-sectional analysis at 24 months and time-to-event analysis suggest an overall pattern of low incidence of bowel and urinary toxic effects in all treatment groups. The QoL instruments used were sensitive to change because acute toxic effects were clearly distinguished using both individual items and bowel, urinary, and sexual domain scores. These acute toxic effects had a small and short-lived effect on general health-related QoL, especially the SF-36 domains of vitality, physical role functioning, and social wellbeing. Overall, changes from baseline to 24 months for urinary, bowel, and most general health-related QoL domains (except role limitations [physical]), were less than previously reported minimally important differences derived from longitudinal anchor-based methods.^17^ Although further development of toxic effects is possible after 2 years, recent studies have reported minimal change in late radiotherapy side-effects after 2 years following external beam radiation therapy.^18^ This suggests that 2 years is an appropriate endpoint for initial PRO reporting.

To our knowledge, this is the first large randomised trial of hypofractionated radiotherapy that used modern radiotherapy techniques to report PROs with follow-up to 24 months. Aluwini and colleagues^19^ reported preliminary results that included PROs up to 3 months in the HYPRO study,^19^ which included 820 patients. Radiotherapy doses were higher in HYPRO (standard fractionation of 39 fractions of 2 Gy in 8 weeks *vs* hypofractionation with 19 fractions of 3·4 Gy in 6·5 weeks) than in CHHiP. Combined clinician-reported outcomes and PROs showed similar acute genitourinary toxic effects between treatments, but increased acute gastrointestinal toxic effects with hypofractionation. A small (124 patients) randomised study of moderate hypofractionation (63 Gy in 20 fractions) versus conventional fractionation (76 Gy in 38 fractions) reported no difference in EPIC scores between treatment groups up to 3 months after radiotherapy.^20^

The PROs in this study are broadly consistent with preliminary data for clinician-reported outcomes in the CHHiP trial,^4^ and the clinician-reported outcomes of a small (168 patients) phase 3 trial in Italy.^21^ Results from another randomised trial that included 203 patients showed a non-significant numerical increase in clinician-reported late gastrointestinal toxic effects with hypofractionation.^22^ However, the radiotherapy dose schedules used in these studies^21^, ^22^ differ substantially from those in CHHiP.

So far, randomised trials^21^, ^22^, ^23^, ^24^, ^25^ reporting the effects of hypofractionation versus conventional fractionation have reported inconsistent results for side-effects and do not clearly show a difference in the rate of increase of genitourinary or gastrointestinal toxic effects between conventional and hypofractionated radiotherapy treatments.^26^ These findings emphasise the need for outcome data from large trials of hypofractionation that use modern radiotherapy techniques. Such studies to compare hypofractionated radiotherapy with standard fractionation, together with the clinician-reported outcomes from CHHiP, will help to confirm whether faecal incontinence, rectal bleeding, or use of urinary pads are more common at a dose of 3 Gy per fraction.

Findings from a trial of hypofractionation^25^ that included 303 patients raised concerns about increased urinary toxic effects after hypofractionated treatment in patients who had compromised urinary function before enrolment, as assessed by clinician-reported LENT and RTOG scores. A formal comparison restricted to patients with baseline dysfunction has not been done in our study, partly because obstructive and irritative symptoms, and consequently overall urinary dysfunction, are not well represented in the UCLA-PCI instrument. We plan to do a formal comparison between groups using clinician-reported outcomes from RTOG and LENT instruments in a separate study. However urinary co-morbidity was well balanced between treatment groups and change in urinary function from baseline did not differ between fractionation schedules. Furthermore, overall urinary bother seems to decrease during the 2 years after radiotherapy, which is consistent with findings from the RT01 dose escalation trial.^27^

Comparison of bowel bother and distress assessed using the UCLA-PCI instrument in both the 74 Gy group of CHHiP and the 74 Gy group of the RT01 trial,^27^ in which conventional radiotherapy planning techniques were used, suggests that patients benefit substantially from improved treatment methods that use intensity-modulated radiotherapy and the dose constraints used in CHHiP. In RT01, 27 (9%) of 289 patients reported moderate bowel bother and nine (3%) patients reported severe bother in the 74 Gy group at 24 months.^28^ This compares with 19 (5%) of 410 patients reporting moderate bother and four (<1%) patients reporting severe bother in the 74 Gy group in CHHiP at 24 months. Similarly, at 24 months, 34 (12%) of 288 patients in RT01^28^ versus 13 (4%) of 312 patients in CHHiP reported moderate bowel distress, and two (<1%) patients in RT01^28^ versus none in CHHiP reported severe bowel distress.

Strengths of our study include the wide age range and large number of patients recruited from different parts of the UK. The use of different QoL instruments was a limitation of the analysis, because it meant that fewer patients reported some important radiotherapy-related toxic effects, including rectal bleeding and faecal incontinence, which were only represented in EPIC. Additionally, domain scores for UCLA-PCI and EPIC are not directly comparable, so separate reporting was necessary with smaller numbers than used for the primary endpoint. EPIC is now regarded as the QoL instrument of choice for localised prostate cancer;^29^ however, our trial was planned before it was widely available. To our knowledge, minimally clinically important differences have not been published for EPIC-50, but will be a valuable addition when available.

Patients might acclimatise to symptoms over time and therefore the most subjective endpoints, such as overall bowel, urinary, and sexual bother, might under-represent the actual toxicity at later timepoints. However, more objective PROs, including rectal bleeding, dysuria, and quality of erections showed similar patterns of toxic effects over time compared with overall bother items, suggesting that overall bother gives a reliable representation of patient experience. Bother items might incorporate a psychosocial component as well as actual functional change, but we believe that overall perception of toxic effects is a comprehensive and comprehensible endpoint in a randomised comparison of PROs.

Patients with missing data at 24 months were more likely to be in a higher NCCN risk group than were those patients with data present. Biochemical recurrence did not exclude patients from the PRO substudy, but patients might have been less willing to complete the questionnaires after relapse. Both NCCN risk group and numbers of patients with missing data did not differ between treatment groups, therefore missing data are unlikely to have substantially biased the randomised comparisons.

PROs at 5 years will be important to confirm our findings. 5-year outcomes, together with longer-term clinician-reported outcomes, will help to elucidate whether late emergent differences exist between treatment groups. 5-year efficacy data from the CHHiP trial will be available in late 2015. Follow-up from complementary randomised studies is ongoing and together these will clarify the role of moderately hypofractionated radiotherapy treatment for localised prostate cancer. Radiotherapy treatments need to balance the potential increased efficacy of biologically increased doses with the risk of increased side-effects. So far, our results show that the bowel and urinary side-effects of moderate hypofractionation for prostate cancer delivered with modern radiotherapy techniques are low and similar to those of standard fractionation.

## Figures and Tables

**Table 1:** Baseline characteristics and clinical history

		**74 Gy in 37 fractions (n=676)**	**60 Gy in 20 fractions (n=686)**	**57 Gy in 19 fractions (n=692)**
Age (years)		69 (65–73)	69 (64–73)	68 (64–73)
T stage				
	T1a/1b/1c/1x	224 (33%)	273 (40%)	252 (36%)
	T2a/b/c/x	393 (58%)	355 (52%)	368 (53%)
	T3a/x	59 (9%)	57 (8%)	71 (10%)
	Unknown	0 (0%)	1 (<1%)	1 (<1%)
Gleason score				
	≤6	248 (37%)	252 (37%)	233 (34%)
	7	406 (60%)	413 (60%)	432 (62%)
	8	22 (3%)	21 (3%)	27 (4%)
Prostate-specific antigen (ng/mL)				
	Median (IQR)	10·4 (7·3–14·6)	11·0 (7·8–15·5)	10·4 (7·2–14·5)
	Mean (SD)	11·3 (5·3)	11·9 (5·8)	11·3 (5·4)
	0·00–4·99	44 (7%)	51 (7%)	46 (7%)
	5·00–9·99	267 (39%)	249 (36%)	274 (40%)
	10·00–19·90	319 (47%)	327 (48%)	321 (46%)
	20·00–40·00	46 (7%)	59 (9%)	49 (7%)
	Unknown	0 (0%)	0 (0%)	2 (<1%)
NCCN risk group				
	Low	104 (15%)	113 (16%)	109 (16%)
	Intermediate	496 (73%)	498 (73%)	496 (72%)
	High	76 (11%)	75 (11%)	87 (13%)
Diabetes				
	Yes	74 (11%)	65 (9%)	77 (11%)
	No	599 (89%)	619 (90%)	606 (88%)
	Unknown	3 (<1%)	2 (<1%)	9 (1%)
Hypertension				
	Yes	248 (37%)	281 (41%)	273 (39%)
	No	423 (63%)	403 (59%)	414 (60%)
	Unknown	5 (<1%)	2 (<1%)	5 (<1%)
Inflammatory bowel or diverticular disease				
	Yes	25 (4%)	21 (3%)	27 (4%)
	No	646 (96%)	663 (97%)	659 (95%)
	Unknown	5 (<1%)	2 (<1%)	6 (1%)
Previous pelvic surgery				
	Yes	55 (8%)	51 (7%)	53 (8%)
	No	616 (91%)	633 (92%)	632 (91%)
	Unknown	5 (<1%)	2 (<1%)	7 (1%)
Symptomatic haemorrhoids in past 12 months				
	Yes	39 (6%)	50 (7%)	52 (8%)
	No	620 (92%)	617 (90%)	623 (90%)
	Unknown	17 (3%)	19 (3%)	17 (2%)
Previous transurethral resection of the prostate				
	Yes	55 (8%)	61 (9%)	62 (9%)
	No	606 (90%)	615 (90%)	617 (89%)
	Unknown	15 (2%)	10 (1%)	13 (2%)
Hormone treatment duration (days)*		140 (113–169)	132 (102–165)	127 (102–157)
Time from androgen suppression to start of radiotherapy (days)		116 (103–138)	118 (103–138)	115 (103–139)
Time from radiotherapy start to end of androgen suppression (days)†		16 (−3 to 42)	6 (−6 to 24)	6 (−7 to 23)

Data are n (%) or median (IQR) unless otherwise stated. NCCN=National Comprehensive Cancer Network.

* Hormone treatment duration was the total duration, including before study entry.

† Negative values are from patients who stopped androgen suppression before starting radiotherapy.

**Table 2:** Bowel, urinary, and sexual domain scores at 24 months for UCLA-PCI and EPIC QoL instruments

	**74 Gy in 37 fractions (n=676)**		**60 Gy in 20 fractions (n=686)**		**57 Gy in 19 fractions (n=692)**		74 Gy *vs* 60 Gy p value*	74 Gy *vs* 57 Gy p value*	60 Gy *vs* 57 Gy p value*
	Number of patients with data	Median (IQR)	Number of patients with data	Median (IQR)	Number of patients with data	Median (IQR)			
Bowel function (UCLA)	312 (46%)	93·8 (82·5–100·0)	310 (45%)	91·8 (79·3–100·0)	331 (48%)	93·8 (81·3–100·0)	0·064	0·77	0·12
Urinary function (UCLA)	311 (46%)	100·0 (81·8–100·0)	313 (46%)	100·0 (81·8–100·0)	334 (48%)	100·0 (83·5–100·0)	0·69	0·47	0·74
Sexual function (UCLA)	307 (45%)	27·1 (4·1–53·1)	300 (44%)	23·4 (7·3–57·3)	321 (46%)	26·0 (7·3–56·3)	0·39	0·33	0·92
Bowel function (EPIC)	95 (14%)	96·4 (89·3–96·4)	94 (14%)	96·4 (89·3–100·0)	100 (14%)	92·9 (85·7–98·2)	0·15	0·51	0·059
Bowel bother (EPIC)	95 (14%)	95·8 (87·5–100·0)	96 (14%)	95·8 (83·3–100·0)	101 (15%)	95·8 (79·2–100·0)	0·54	0·15	0·38
Bowel summary (EPIC)	93 (14%)	94·2 (88·5–98·1)	94 (14%)	94·2 (87·5–100·0)	99 (14%)	94·2 (84·6–98·1)	0·41	0·41	0·15
Urinary function (EPIC)	97 (14%)	100·0 (93·4–100·0)	99 (14%)	100·0 (88·4–100·0)	104 (15%)	100·0 (90·9–100·0)	0·18	0·84	0·11
Urinary bother (EPIC)	89 (13%)	89·3 (79·2–96·4)	95 (14%)	89·3 (78·6–96·4)	102 (15%)	90·5 (75·0–96·4)	0·64	0·72	0·38
Urinary summary (EPIC)	89 (13%)	91·0 (85·4–97·9)	95 (14%)	93·1 (82·7–97·9)	101 (15%)	93·8 (82·7–97·9)	1·00	0·91	0·93
Sexual function (EPIC)	89 (13%)	21·6 (0·0–60·0)	88 (13%)	21·6 (0·0–53·4)	98 (14%)	26·6 (0·0–58·4)	0·78	0·74	0·53
Sexual summary (EPIC)	92 (14%)	28·4 (15·2–62·5)	93 (14%)	23·7 (16·7–58·3)	99 (14%)	27·8 (13·8–61·2)	0·60	0·84	0·74

EPIC-50 was used for bowel and urinary domains and EPIC-26 for sexual domains. UCLA-PCI=University of California, Los Angeles Prostate Cancer Index. EPIC=Expanded Prostate Cancer Index Composite.

* Mann-Whitney *U* test.
