# Supplementary material for: Hypofractionated radiotherapy versus conventionally fractionated radiotherapy for patients with intermediate-risk localised prostate cancer: 2-year patient-reported outcomes of the randomised, non-inferiority, phase 3 CHHiP trial
Source: Lancet Oncol. 2015 Dec;16(16):1605–16. doi: 10.1016/S1470-2045(15)00280-6 (PMC4664817; doi:10.1016/S1470-2045(15)00280-6)
Supplement: Supplementary appendix [file mmc1.pdf]

THE LANCET Oncology

Supplementary appendix

This appendix formed part of the original submission and has been peer reviewed. We post it as supplied by the authors.

Supplement to: Wilkins A, Mossop H, Syndikus I, et al. Hypofractionated radiotherapy versus conventionally fractionated radiotherapy for patients with intermediate-risk localised prostate cancer: 2-year patient-reported outcomes of the randomised, non-inferiority, phase 3 CHHiP trial. *Lancet Oncol* 2015; published online Oct 28. http://dx.doi.org/10.1016/S1470-2045(15)00280-6.

APPENDICES

Table 1: Baseline characteristics of patients missing versus present from QoL analysis

	Present (n=2054)		Missing (n=46)	
	n	%	n	%
**Age in years (median)**	68.6		68.4	
	Wilcoxon rank-sum p=0.71			
**T-stage**				
T1a/1b/1c/1x	749	36.5%	22	47.8%
T2a/b/c/x	1,116	54.3%	17	37.0%
T3a/x	187	9.1%	7	15.2%
Unknown	2	0.1%	0	0.0%
	Chi^2: p trend=0.57			
**Gleason score**				
≤6	733	35.7%	15	32.6%
7	1,250	60.9%	30	65.2%
8	70	3.4%	1	2.2%
	Fishers exact test=0.91			
**Categorised PSA**				
0-4.99	141	6.9%	5	10.9%
5-9.99	790	38.5%	17	37.0%
10-19.9	967	47.1%	19	41.3%
20-49.99	154	7.5%	5	10.9%
Unknown	2	0.1%	0	0.0%
	Chi^2: p trend =0.78			
	Kruskall Wallis (PSA continuous variable) p=0.87			
**NCCN Risk Group**				
Low	326	15.9%	7	15.2%
Intermediate	1,490	72.5%	29	63.0%
High	238	11.6%	10	21.7%
	Chi^2: p trend =0.17			
**Diabetes**				
Yes	216	10.5%	6	13.0%
No	1,824	88.9%	39	84.8%
Unknown	14	0.6%	1	2.2%
	Fisher's exact test: p=0.20			
**Hypertension**				
Yes	802	39.0%	23	50.0%
No	1,240	60.4%	23	50.0%
Unknown	12	0.6%	0	0.0%
	Fisher's exact test: p=0.34			
**Inflammatory Bowel or Diverticular disease**				
Yes	73	3.6%	3	6.5%
No	1,968	95.8%	43	93.5%
Unknown	13	0.6%	0	0.0%
	Fisher's exact test: p=0.39			
**Previous pelvic surgery**				
Yes	159	7.7%	1	2.2%
No	1,881	91.6%	45	97.8%
Unknown	14	0.7%	0	0.0%
	Fisher's exact test: p=0.43			
**Symptomatic haemorrhoids in past year**				
Yes	141	6.9%	3	6.5%
No	1,860	90.6%	43	93.5%
Unknown	53	2.6%	0	0.0%
	Fisher's exact test: p=0.83			
**Any previous TURP**				
Yes	178	8.7%	2	4.3%
No	1,838	89.5%	44	95.7%
Unknown	38	1.9%	0	0.0%
	Fisher's exact test: p=0.57			

Table 2: Baseline characteristics of patients missing versus present from 24 months follow up within QoL analysis

	Present (n=1272)		Missing (n=828)	
	n	%	n	%
**Age in years (median)**	68.3		69.2	
	**Wilcoxon rank-sum p=0.01**			
**T-stage**				
T1a/1b/1c/1x	497	39.1%	274	33.1%
T2a/b/c/x	668	52.5%	465	56.2%
T3a/x	107	8.4%	87	10.5%
Unknown	0	0.0%	2	0.2%
	**Chi^2: p trend=0.004**			
**Gleason score**				
≤6	469	37.0%	279	33.7%
7	765	60.2%	515	62.2%
8	37	2.9%	34	4.1%
	**Chi^2: p trend=0.07**			
**Categorised PSA**				
0-4.99	94	7.4%	52	6.3%
5-9.99	503	39.5%	304	36.7%
10-19.9	581	45.7%	405	48.9%
20-49.99	92	7.2%	67	8.1%
Unknown	2	0.1%	0	0.0%
	**Chi^2: p trend =0.07**			
**PSA (continuous)**	**Kruskall Wallis p=0.06**			
**NCCN Risk Group**				
Low	235	18.5%	98	11.8%
Intermediate	912	71.7%	607	73.3%
High	125	9.8%	123	14.9%
	**Chi^2: p trend =<0.0001**			
**Diabetes**				
Yes	120	9.4%	102	12.3%
No	1,142	89.8%	721	87.1%
Unknown	10	0.9%	5	0.6%
	**Fisher's exact test: p=0.10**			
**Hypertension**				
Yes	486	38.2%	339	41.0%
No	777	61.1%	486	58.7%
Unknown	9	0.7%	3	0.4%
	**Fisher's exact test: p=0.24**			
**Inflammatory Bowel or Diverticular disease**				
Yes	42	3.3%	34	4.1%
No	1,221	96.0%	790	95.4%
Unknown	9	0.7%	4	0.5%
	**Fisher's exact test: p=0.56**			
**Previous pelvic surgery**				
Yes	96	7.5%	64	7.7%
No	1,164	91.5%	762	92.0%
Unknown	12	0.9%	2	0.2%
	**Fisher's exact test: p=0.08**			
**Symptomatic haemorrhoids in past year**				
Yes	87	6.8%	57	6.9%
No	1,152	90.6%	751	90.7%
Unknown	33	2.6%	20	2.4%
	**Chi^2: p=0.93**			
**Any previous TURP**				
Yes	116	9.1%	64	7.7%
No	1,132	88.9%	750	90.6%
Unknown	24	1.8%	14	1.7%
	**Chi^2: p=0.45**			

Table 3a: Cross sectional analysis of bowel side effects at 2 years

E: EPIC QoL instrument, U: UCLA-PCI QoL instrument

Item assessed	Severity	Side effects at 2 years post radiotherapy									60Gy vs 74Gy	57Gy vs 74Gy	60Gy vs 57Gy
		74Gy			60Gy			57Gy			Chi-squared p_{trend}	Chi-squared p_{trend}	Chi-squared p_{trend}
		n	total	% (99% CI)	n	total	% (99% CI)	n	total	% (99% CI)			
**BOWEL ENDPOINTS**													
Overall bowel bother (E & U)	Small problem	26	410	6.3% (4.2-9.2)	28	411	6.8% (4.6-9.7)	38	437	8.7% (6.2-11.7)			
	Moderate problem	19	410	4.6% (2.8-7.1)	23	411	5.6% (3.6-8.3)	21	437	4.8% (3.0-7.3)			
	Big problem	4	410	1.0% (0.3-2.5)	3	411	0.7% (0.2-2.1)	3	437	0.7% (0.1-2.0)	0.64	0.59	0.95
Rectal urgency (E and U)	> once per week	16	406	3.9% (2.3-6.3)	17	406	4.2% (2.5-6.6)	17	433	3.9% (2.3-6.2)			
	Once a day	17	406	4.2% (2.5-6.6)	27	406	6.7% (4.4-9.5)	27	433	6.2% (4.1-8.9)			
	> once a day	8	406	2.0% (0.9-3.8)	9	406	2.2% (1.0-4.2)	11	433	2.5% (1.3-4.5)	0.48	0.42	0.93
Faecal incontinence (E only)	> once per week	3	95	3.2% (0.7-9.0)	0	97	0.0% (0-3.7)	8	101	7.9% (3.5-15.0)			
	Once a day	0	95	0.0% (0-3.8)	3	97	3.1% (0.6-8.8)	3	101	3.0% (0.6-8.4)			
	> once a day	1	95	1.1% (0.03-5.7)	1	97	1.0% (0.03-5.6)	0	101	0.0% (0.0-3.6)	0.16	0.02	0.26
Rectal bleeding (E only)	Half the time	3	95	3.2% (0.7-9.0)	1	97	1.0% (0.03-5.6)	2	101	2.0% (0.2-6.8)			
	Usually	1	95	1.1% (0.03-5.7)	1	97	1.0% (0.03-5.6)	0	101	0.0% (0-3.6)			
	Always	0	95	0% (0-3.8)	1	97	1.0% (0.03-5.6)	2	101	2.0% (0.2-7.0)	0.61	0.71	0.37
Loose or liquid stools (E and U)	Half the time	36	406	8.9% (6.3-12.1)	58	407	14.3% (11.0-18.0)	55	432	12.7% (9.7-16.2)			
	Usually	16	406	3.9% (2.3-6.3)	14	407	3.4% (1.9-5.7)	13	432	3.0% (1.6-5.1)			
	Always	3	406	0.7% (0.2-2.1)	3	407	0.7% (0.2-2.1)	4	432	0.9% (0.3-2.4)	0.30	0.40	0.81
Daily bowel movements (E only)	3-4	12	97	12.4% (6.6-20.6)	9	96	9.4% (4.4-17.1)	16	103	15.5% (9.1-24.0)			
	>4	1	97	1.0% (0.03-5.6)	2	96	2.1% (0.3-7.3)	2	103	1.9% (0.2-6.8)	0.68	0.43	0.23
Crampy abdominal pain (E and U)	> once per week	19	410	4.6% (2.8-7.1)	17	408	4.2% (2.4-6.6)	20	435	4.6% (2.8-7.0)			
	Once a day	6	410	1.5% (0.5-3.2)	5	408	1.2% (0.4-2.8)	8	435	1.8% (0.8-3.6)			
	More than once a day	3	410	0.7% (0.2-2.1)	5	408	1.2% (0.4-2.8)	5	435	1.1% (0.4-2.7)	0.76	0.44	0.64
Bowel distress (U only)	A little	64	312	20.5% (16.2-25.4)	65	310	21.0% (16.6-26.0)	70	331	21.1% (16.9-26.0)			
	Moderate	13	312	4.2% (2.2-7.0)	21	310	6.8% (4.2-10.1)	11	331	3.3% (1.7-5.9)			
	Severe	0	312	0% (0.0-1.2)	1	310	0.3% (<0.01-1.8)	2	331	0.6% (0.07-2.2)	0.17	0.97	0.17

Table 3b: Cross sectional analysis of urinary and sexual side effects at 2 years

E: Epic QoL instrument, U: UCLA-PCI QoL instrument

Item assessed	Severity	Side effects at 2 years post radiotherapy									60Gy vs 74Gy	57Gy vs 74Gy	60Gy vs 57Gy
		74Gy			60Gy			57Gy			Chi-squared p_{trend}	Chi-squared p_{trend}	Chi-squared p_{trend}
		n	total	% (99% CI)	n	total	% (99% CI)	n	total	% (99% CI)			
**URINARY ENDPOINTS**													
Overall urinary bother (E and U)	Small problem	27	406	6.7% (4.4-9.5)	35	410	8.5% (6.0-11.7)	35	436	8.0% (5.7-11.0)			
	Moderate problem	20	406	4.9% (3.0-7.5)	17	410	4.1% (2.4-6.6)	20	436	4.6% (2.8-7.0)			
	Big problem	1	406	0.2% (<0.01-1.4)	5	410	1.2% (0.4-2.8)	5	436	1.1% (0.4-2.7)	0.31	0.72	0.51
Urinary control (E and U)	Occasional dribble	136	407	33.4% (28.8-38.2)	125	412	30.3% (26.0-35.0)	143	437	32.7% (28.3-37.3)			
	Frequent dribble	10	407	2.5% (1.2-4.5)	16	412	3.9% (2.2-6.2)	4	437	0.9% (0.2-2.3)			
	No control whatsoever	1	407	0.2% (<0.01-1.4)	4	412	1.0% (0.3-2.5)	1	437	0.2% (<0.01-1.3)	0.75	0.29	0.18
Use of urinary pads (E and U)	1-2 pads per day	12	404	3.0% (1.5-5.1)	12	407	2.9% (1.5-5.1)	10	435	2.3% (1.1-4.2)			
	≥3 pads per day	0	404	0.0% (0-0.9)	1	407	0.2% (<0.01-1.4)	1	435	0.2% (<0.01-1.3)	0.85	0.70	0.56
Haematuria (E only)	> once per week	0	97	0.0% (0-5.3)	0	99	0.0% (0-5.2))	0	104	0.0% (0-5.0)			
	Once a day	0	97	0.0% (0-5.3)	0	99	0.0% (0-5.2)	0	104	0.0% (0-5.0)			
	> once a day	0	97	0.0% (0-5.3)	2	99	2.0% (0.1-9.0)	0	104	0.0% (0-5.0)	0.08	0.17	0.61
Dysuria (E only)	> once per week	1	97	1.0% (0.03-5.6)	2	98	2.0% (0.2-7.2)	0	104	0.0% (0.0-3.5%)			
	Once a day	0	97	0.0% (0.0-3.7)	2	98	2.0% (0.2-7.2)	1	104	1.0% (0.02-5.2)			
	> once a day	2	97	2.1% (0.3-7.3)	2	98	2.0% (0.2-7.2)	2	104	1.9% (0.2-6.8)	0.28	0.62	0.12
**SEXUAL ENDPOINTS**													
Overall sexual bother (E and U)	Small problem	52	393	13.2% (10.0-17.0)	61	396	15.4% (12.0-19.3)	69	416	16.6% (13.1-20.5)			
	Moderate problem	70	393	17.8% (14.2-22.0)	64	396	16.2% (12.7-20.2)	66	416	15.9% (12.5-19.7)			
	Big problem	77	393	19.6% (15.8-23.9)	90	396	22.7% (18.7-27.2)	91	416	21.9% (18.0-26.2)	0.47	0.50	0.95
Quality of erections (E and U)	Limited sexual activity	77	400	19.3% (15.5-23.5)	86	392	21.9% (17.9-26.4)	94	420	22.4% (18.5-26.7)			
	No sexual activity	89	400	22.3% (18.3-26.6)	94	392	24.0% (19.8-28.5)	100	420	23.8% (19.8-28.2)			
	None at all	123	400	30.8% (26.3-35.5)	109	392	27.8% (23.4-32.5)	112	420	26.7% (22.5-31.2)	0.75	0.46	0.68
Frequency of erections (E and U)	Erection 50% times desired	49	395	12.4% (9.3-16.1)	38	385	9.9% (7.1-13.3)	44	417	10.6% (7.8-13.9)			
	Erection <50% times desired	46	395	11.6% (8.7-15.2)	54	385	14.0% (10.7-17.9)	71	417	17.0% (13.5-21.0)			
	Never when desired	188	395	47.6% (42.6-52.6)	184	385	47.8% (42.7-52.9)	181	417	43.4% (38.6-48.3)	0.80	0.70	0.53
Morning erections (U only)	Not often	47	308	15.3% (11.4-19.8)	43	302	14.2% (10.5-18.7)	49	323	15.2% (11.4-19.6)			
	Seldom	80	308	26.0% (21.2-31.3)	87	302	28.8% (23.8-34.3)	106	323	32.8% (27.7-38.2)			
	Never	148	308	48.1% (42.4-53.8)	137	302	45.4% (40.0-51.2)	132	323	40.9% (35.5-46.4)	0.54	0.37	0.82

Table 4: Emergent cumulative bowel, urinary and sexual side effects to 2 years (E: EPIC QoL instrument, U: UCLA-PCI QoL instrument)

Item assessed	Severity	Number with symptom prior to radiotherapy	Emergent cumulative side effects at 2 years								
			74Gy			60Gy			57Gy		
			n	total	% (99% CI)	n	total	% (99% CI)	n	total	% (99% CI)
**BOWEL ENDPOINTS**											
Overall bowel bother (E & U)	Small or worse	162	138	596	23.2% (19.8-26.8)	141	577	24.4% (21.0-28.2)	126	589	21.4% (18.1-24.9)
	Moderate or worse	68	75	622	12.1% (9.6-14.9)	75	614	12.2% (9.7-15.1)	63	620	10.2% (7.9-12.8)
Rectal urgency (E and U)	>Once per week	228	143	563	25.4% (21.9-29.2)	144	568	25.4% (21.8-29.1)	134	562	23.8% (20.4-27.6)
	≥Once a day	164	107	582	18.4% (15.3-21.8)	107	584	18.3% (15.3-21.7)	100	591	16.9% (14.0-20.2)
Faecal incontinence (E only)	>Once per week	7	10	179	5.6% (2.7-10.0)	18	186	9.7% (5.8-14.9)	20	195	10.3% (6.4-15.4)
	≥Once a day	5	2	180	1.1% (0.1-4.0)	12	186	6.5% (3.4-11.0)	9	196	4.6% (2.1-8.5)
Rectal bleeding (E only)	Half the time or worse	7	7	178	3.9% (1.6-7.9)	17	187	9.1% (5.4-14.2)	11	195	5.6% (2.8-9.9)
	Usually or worse	3	2	180	1.1% (0.1-4.0)	8	187	4.3% (1.9-8.3)	5	197	2.5% (0.8-5.8)
Loose or liquid stools (E and U)	Half the time or worse	238	139	556	25.0% (21.5-28.8)	142	561	25.3% (21.8-29.1)	155	567	27.3% (23.7-31.2)
	Usually or worse	66	54	616	8.8% (6.7-11.3)	67	618	10.8% (8.5-13.6)	63	622	10.1% (7.9-12.8)
Daily bowel movements (E only)	≥3-4	23	27	177	15.3% (10.3-21.4)	34	180	18.9% (13.5-25.4)	40	189	21.2% (15.6-27.7)
	>4	1	4	182	2.2% (0.6-5.5)	5	189	2.6% (0.9-6.1)	4	197	2.0% (0.6-5.1%)
Crampy abdominal pain (E and U)	>Once per week	115	71	610	11.6% (9.2-14.5)	69	588	11.7% (9.2-14.6)	87	610	14.3% (11.6-17.3)
	≥Once a day	62	31	624	5.0% (3.4-7.0)	42	610	6.9% (5.0-9.2)	46	627	7.3% (5.4-9.7)
Bowel distress (U only)	A little or worse	300	164	407	40.3% (35.5-45.2)	161	395	40.8% (35.9-45.8)	136	406	33.5% (28.9-38.3)
	Moderate or worse	58	61	485	12.6% (9.8-15.9)	53	474	11.2% (8.5-14.4)	41	491	8.4% (6.1-11.2)
**URINARY ENDPOINTS**											
Overall urinary bother (E and U)	Small or worse	363	100	517	19.3% (16.0-23.0)	102	521	19.6% (16.3-23.2)	86	522	16.5% (13.3-20.0)
	Moderate or worse	139	57	599	9.4% (7.2-12.1)	55	584	9.4% (7.2-12.1)	51	601	8.5% (6.4-11.0)
Urinary control (E and U)	Occasional dribble or worse	551	153	448	34.2% (30.8-38.7)	153	465	32.9% (28.6-37.4)	138	458	30.1% (26.0-34.6)
	Frequent dribble or worse	40	33	632	5.2% (3.6-7.3)	41	619	6.6% (4.8-8.9)	20	631	3.2% (1.9-4.9)
Use of urinary pads (E and U)	≥1-2 pads per day	32	25	626	4.0% (2.6-5.8)	25	624	4.0% (2.6-5.8)	15	636	2.4% (1.3-3.9)
	≥3 pads per day	2	2	639	0.3% (0.04-1.1)	5	632	0.8% (0.3-1.8)	5	645	0.8% (0.3-1.8)
Haematuria (E only)	>Once per week	5	0	179	0.0% (0.0-2.0)	6	187	3.2% (1.2-6.9)	1	198	0.5% (0.01-2.8)
	≥Once a day	4	0	180	0.0% (0.0-2.0)	5	187	2.7% (0.9-6.1)	1	198	0.5% (0.01-2.8)
Dysuria (E only)	>Once per week	16	11	174	6.3% (3.2-11.0)	22	184	12.0% (7.6-17.5)	13	192	6.8% (3.7-11.3)
	≥Once a day	12	9	176	5.1% (2.4-9.5)	13	184	7.1% (3.8-11.8)	10	194	5.2% (2.5-9.3)
**SEXUAL ENDPOINTS**											
Overall sexual bother (E and U)	Small or worse	888	187	329	56.8 (51.3%-62.3)	196	324	60.5% (54.9-65.9)	202	344	58.7% (53.3-64.0)
	Moderate or worse	690	182	404	45.0% (40.1-50.0)	170	386	44.0% (39.0-49.2)	179	405	44.2% (39.3-49.2)
Quality of erections (E and U)	Limited sexual activity or worse	1208	149	214	69.6% (0.63-0.76)	166	227	73.1% (66.9-78.8)	167	235	71.1% (64.8-76.8)
	No sexual activity	1050	130	264	49.2% (43.1-55.4)	139	279	49.8% (43.8-55.8)	148	291	50.9% (50.0-56.7)
Frequency of erections (E and U)	Erection ≤50% times desired	1220	152	211	72.0% (65.5-80.0)	148	217	68.2% (61.6-74.3)	154	221	69.7% (63.2-75.7)
	Erection <50% times desired	1111	132	234	56.4% (49.8-62.9)	148	261	56.7% (50.5-62.8)	147	263	55.9% (49.7-62.0)
Morning erections (U only)	<50% of the time	1133	92	106	86.8% (78.8-92.6)	107	117	91.5% (84.8-95.8)	120	127	94.5% (89.0-97.8)
	<25% of the time	1051	95	127	74.8% (66.3-82.1)	102	144	70.8% (62.7-78.1)	125	161	77.6% (70.4-83.8)

Table 5: Survival analysis to show time to small* or worse and moderate* or worse events for all bowel, urinary and sexual endpoints.

Item assessed	Severity	Total number of patients	Total number of events	60Gy vs 74Gy		57Gy vs 74Gy		60Gy vs 57Gy	
				HR (99% CI)	p-value	HR (99% CI)	p-value	HR (99% CI)	p-value
**BOWEL ENDPOINTS**									
Overall bowel bother (E & U)	Small or worse	1762	405	1.1 (0.80-1.48)	0.49	0.90 (0.65-1.24)	0.39	0.83 (0.60-1.13)	0.12
	Moderate or worse	1856	213	1.02 (0.67-1.56)	0.90	0.85 (0.54-1.31)	0.34	0.83 (0.53-1.28)	0.25
Rectal urgency (E and U)	>Once per week	1693	421	1.04 (0.77-1.41)	0.74	0.94 (0.69-1.28)	0.65	0.90 (0.66-1.23)	0.37
	≥Once a day	1757	314	1.03 (0.72-1.47)	0.83	0.93 (0.65-1.33)	0.62	0.90 (0.63-1.29)	0.43
Faecal incontinence (E only)	>Once per week	560	48	1.79 (0.65-4.95)	0.14	1.94 (0.71-5.26)	0.07	1.08 (0.47-2.50)	0.81
	≥Once a day	562	23	5.92 (0.83-42.3)	0.01	4.38 (0.58-32.8)	0.03	0.74 (0.24-2.30)	0.49
Rectal bleeding (E only)	Half the time or worse	560	35	2.37 (0.75-7.55)	0.05	1.52 (0.44-5.27)	0.39	0.64 (0.24-1.73)	0.24
	Usually or worse	564	15	3.82 (0.81-18.0)	0.07	2.38 (0.46-12.3)	0.28	0.62 (0.14-2.71)	0.38
Loose or liquid stools (E and U)	Half the time or worse	1684	436	1.05 (0.78-1.43)	0.64	1.13 (0.84-1.53)	0.30	1.07 (0.79-1.45)	0.54
	Usually or worse	1856	184	1.28 (0.80-2.05)	0.17	1.18 (0.73-1.90)	0.37	0.92 (0.59-1.45)	0.64
Daily bowel movements (E only)	≥3-4	546	101	1.23 (0.63-2.39)	0.44	1.41 (0.74-2.67)	0.16	1.14 (0.63-2.09)	0.56
	>4	568	13	1.19 (0.21-6.72)	0.79	0.92 (0.15-5.69)	0.91	0.77 (0.14-4.34)	0.70
Crampy abdominal pain (E and U)	>Once per week	1808	227	1.04 (0.67-1.61)	0.82	1.26 (0.83-1.90)	0.15	1.21 (0.80-1.83)	0.24
	≥Once a day	1861	119	1.44 (0.78-2.65)	0.12	1.51 (0.83-2.74)	0.08	1.05 (0.60-1.81)	0.81
Bowel distress (U only)	A little or worse	1208	461	1.07 (0.80-1.42)	0.57	0.80 (0.60-1.08)	0.06	0.75 (0.56-1.01)	0.01
	Moderate or worse	1450	155	0.89 (0.55-1.45)	0.55	0.65 (0.38-1.09)	0.03	0.72 (0.42-1.23)	0.12
**URINARY ENDPOINTS**									
Overall urinary bother (E and U)	Small or worse	1560	288	1.03 (0.72-1.48)	0.85	0.85 (0.58-1.24)	0.28	0.83 (0.57-1.21)	0.20
	Moderate or worse	1784	163	1.02 (0.63-1.66)	0.93	0.90 (0.55-1.48)	0.61	0.88 (0.53-1.45)	0.51
Urinary control (E and U)	Occasional dribble or worse	1371	444	0.98 (0.73-1.32)	0.89	0.87 (0.64-1.17)	0.22	0.88 (0.65-1.91)	0.28
	Frequent dribble or worse	1882	94	1.32 (0.72-2.41)	0.23	0.62 (0.30-1.28)	0.09	0.47 (0.23-0.95)	0.00
Use of urinary pads (E and U)	≥1-2 pads per day	1886	65	1.03 (0.50-2.13)	0.92	0.60 (0.26-1.40)	0.11	0.59 (0.25-1.36)	0.10
	≥3 pads per day	1916	12	2.61 (0.30-22.5)	0.24	2.49 (0.29-21.5)	0.25	0.95 (0.19-4.87)	0.94
Haematuria (E only)	>Once per week	564	7	N/A		N/A		0.16 (0.01-2.61)	0.05
	≥Once a day	565	6	N/A		N/A		0.19 (0.01-3.25)	0.09
Dysuria (E only)	>Once per week	550	46	1.96 (0.76-5.08)	0.06	1.12 (0.39-3.22)	0.76	0.57 (0.23-1.41)	0.10
	≥Once a day	554	32	1.40 (0.46-4.28)	0.44	1.05 (0.32-3.42)	0.91	0.75 (0.25-2.21)	0.48
**SEXUAL ENDPOINTS**									
Overall sexual bother (E and U)	Small or worse	997	585	1.19 (0.92-1.55)	0.09	1.14 (0.88-1.48)	0.17	0.96 (0.74-1.24	0.63
	Moderate or worse	1195	531	1.01 (0.77-1.33)	0.94	1.04 (0.80-1.37)	0.70	1.03 (0.78-1.36)	0.78
Quality of erections (E and U)	Limited sexual activity or worse	676	482	1.21 (0.90-1.62)	0.08	1.16 (0.87-1.55)	0.22	0.96 (0.72-1.27)	0.75
	No sexual activity	834	417	1.05 (0.76-1.43)	0.69	1.08 (0.79-1.47)	0.57	1.03 (0.76-1.39)	0.79
Frequency of erections (E and U)	Erection ≤50% times desired	649	454	0.97 (0.72-1.31)	0.82	1.06 (0.79-1.42)	0.70	1.09 (0.81-1.46)	0.45
	Erection <50% times desired	758	427	1.04 (0.76-1.41)	0.70	1.03 (0.75-1.40)	0.93	0.99 (0.73-1.34)	0.95
Morning erections (U only)	<50% of the time	350	319	1.09 (0.76-1.58)	0.54	1.13 (0.79-1.61)	0.41	1.03 (0.73-1.45)	0.81
	<25% of the time	432	322	0.90 (0.63-1.31)	0.48	1.03 (0.73-1.46)	0.81	1.14 (0.81-1.61)	0.32

***** For 5 part scales “small” was a score of 3 out of 5 and “moderate” a score of 4 out of 5. For 4 part scales “small” was a score of 2 out of 4 and “moderate” a score of 3 out of 4. For 3 part scales “small” was a score of 2 out of 3 and “moderate” was a score of 3 out of 3.
E: EPIC QoL instrument, U: UCLA-PCI QoL instrument, HR: Hazard ratio, N/A: Not assessable

Table 6: Ordinal logistic regression to show odds of an increase in score in experimental arm versus control arm from baseline or pre-radiotherapy* to 24 months after radiotherapy.

*For bowel and urinary endpoints, to maximise numbers the pre-radiotherapy score was used as a surrogate baseline score unless missing in which case the baseline score was used (exact numbers shown below†). For sexual endpoints only the baseline score at trial entry was used.
N/A: Not assessable, E: EPIC QoL instrument, U: UCLA-PCI QoL instrument
Note some haematuria OR are not shown as non-assessable due to low event rates.

Item assessed	Numbers assessed	60Gy vs 74Gy		57Gy vs 74Gy		60Gy vs 57Gy	
		OR (99% CI)	p-value	OR (99% CI)	p-value	OR (99% CI)	p-value
**BOWEL ENDPOINTS**							
Overall bowel bother (E & U)	1056	0.85 (0.57-1.26)	0.29	0.84 (0.57-1.24)	0.25	0.99 (0.67-1.46)	0.95
Rectal urgency (E and U)	1044	1.08 (0.71-1.65)	0.62	1.07 (0.70-1.62)	0.69	0.99 (0.65-1.50)	0.94
Faecal incontinence (E only)	168	5.64 (0.7-44.2)	0.03	3.49 (0.72-16.9)	0.04	1.07 (0.32-3.63)	0.90
Rectal bleeding (E only)	168	1.61 (0.42-6.21)	0.36	1.10 (0.32-3.82)	0.84	0.69 (0.17-2.72)	0.49
Loose or liquid stools (E and U)	1046	1.16 (0.80-1.69)	0.31	1.03 (0.71-1.48)	0.85	0.89 (0.61-1.29)	0.41
Daily bowel movements (E only)	171	0.85 (0.17-4.38)	0.80	1.38 (0.31-6.09)	0.83	1.77 (0.33-9.64)	0.38
Crampy abdominal pain (E and U)	1052	1.41 (0.81-2.46)	0.11	0.99 (0.58-1.67)	0.95	0.72 (0.42-1.21)	0.10
Bowel distress (U only)	814	1.07 (0.65-1.76)	0.72	0.90 (0.56-1.46)	0.57	0.84 (0.52-1.37)	0.36
**URINARY ENDPOINTS**							
Overall urinary bother (E and U)	1053	1.30 (0.89-1.91)	0.07	0.99 (0.68-1.43)	0.92	0.76 (0.52-1.10)	0.06
Urinary control (E and U)	1054	1.01 (0.64-1.58)	0.97	0.77 (0.49-1.21)	0.14	0.77 (0.49-1.21)	0.14
Use of urinary pads (E and U)	1044	1.69 (0.44-6.47)	0.32	1.21 (0.37-3.91)	0.68	0.75 (0.20-2.78)	0.57
Haematuria (E only)	174	1.48 (0.13-16.38)	0.67	NA	NA	2.00 (0.20-19.61)	0.44
Dysuria (E only)	174	2.06 (0.31-13.61)	0.32	1.01 (0.21-4.86)	0.98	0.49 (0.07-3.20)	0.32
**SEXUAL ENDPOINTS**							
Overall sexual bother (E and U)	446	0.70 (0.41-1.20)	0.09	0.91 (0.53-1.55)	0.64	1.27 (0.74-1.28)	0.25
Quality of erections (E and U)	447	1.59 (0.91-2.76)	0.03	1.15 (0.66-2.01)	0.51	0.71 (0.41-1.23)	0.11
Frequency of erections (E and U)	438	1.42 (0.82-2.45)	0.10	1.22 (0.71-2.10)	0.35	0.84 (0.49-1.45)	0.41
Morning erections (U only)	381	1.89 (1.03-3.47)	0.01	1.43 (0.78-2.63)	0.13	0.76 (0.42-1.36)	0.22

† For bowel and urinary endpoints numbers of patients used at baseline (BL) or pre-radiotherapy (PRT): Overall bowel bother PRT 971, BL 85; rectal urgency PRT 959, BL 85; faecal incontinence and rectal bleeding PRT 154, BL 14; loose or liquid stools PRT 961, BL 85; daily bowel movements PRT 159, BL 12; crampy abdominal pain PRT 970, BL 82; bowel distress PRT 748, BL 66; overall urinary bother PRT 969, BL 84; urinary control PRT 972, BL 82; urinary pads PRT 958, BL 86; haematuria and dysuria PRT 162, BL 12.

Table 7: General quality of life domain scores measured by FACT-P, SF12 and SF36 instruments at 2 years
***Mann-Whitney U test**
Note for all 3 QoL instruments, high scores indicate a better quality of life than low scores

Domain score at 2 years	74 Gy		60 Gy		57 Gy		74Gy vs 60Gy p-value*	74Gy vs 57Gy p-value*	60Gy vs 57Gy p-value*
	N	Median (IQR)	N	Median (IQR)	N	Median (IQR)			
**FACT-P (maximum score 28)**									
Physical wellbeing	310	26.0 (25.0-27.0)	311	26.0 (24.0-27.0)	331	26.0 (24.0-27.0)	0.57	0.89	0.63
Social/family wellbeing	311	24.0 (21.0-26.0)	311	24.0 (20.0-26.8)	330	24.0 (20.0-26.0)	0.23	0.17	0.83
Emotional wellbeing	308	22.5 (20.0-24.0)	308	22.0 (20.0-24.0)	329	22.8 (20.0-24.0)	0.02	0.44	0.11
Functional wellbeing	308	25.0 (22.0-27.0)	308	25.0 (21.0-27.0)	329	25.0 (21.0-27.0)	0.75	0.28	0.53
**SF12 (maximum score 100)**									
Mental health composite	90	71.9 (63.3-76.5)	90	69.1 (60.9-75.2)	99	71.4 (61.6-75.7)	0.07	0.28	0.42
Physical health composite	90	71.1 (63.9-75.2)	90	70.2 (57.8-74.2)	99	68.4 (55.5-73.1)	0.33	0.04	0.35
**SF36 (maximum score 100)**									
Physical functioning	312	90.0 (69.4-95.0)	311	90.0 (75.0-100.0)	333	90.0 (70.0-95.0)	0.25	0.34	0.79
Role limitations (physical)	312	75.0 (0.0-100.0)	313	100.0 (0.0-100.0)	333	75.0 (0.0-100.0)	0.30	0.94	0.26
Role limitations (emotional)	308	100.0 (66.7-100.0)	313	100.0 (66.7-100.0)	335	100.0 (33.3-100.0)	0.93	0.08	0.09
Vitality	307	68.8 (50.0-75.0)	313	68.8 (50.0-75.0)	335	68.8 (50.0-81.25)	0.55	0.40	0.86
Emotional wellbeing	307	85.0 (75.0-95.0)	312	85.0 (70.0-94.4)	334	85.0 (70.0-90.0)	0.32	0.09	0.51
Social functioning	313	100.0 (87.5-100.0)	316	100.0 (75.0-100.0)	339	100.0 (75.0-100.0)	0.40	0.37	0.97
Pain	310	90.0 (67.5-100.0)	314	90.0 (67.5-100.0)	333	90.0 (67.5-100.0)	0.76	0.85	0.60
General health	290	75.0 (62.5-87.5)	302	75.0 (60.0-87.5)	318	75.0 (56.3-87.5)	0.83	0.63	0.53

Figure 1: EPIC domain scores for bowel function † (A), bowel bother † (B), urinary function † (C), urinary bother † (D) and sexual function (E). Note EPIC-50 was used for bowel and urinary domains and EPIC-26 was used for sexual domain scores therefore sexual bother is a single item reported in main manuscript.

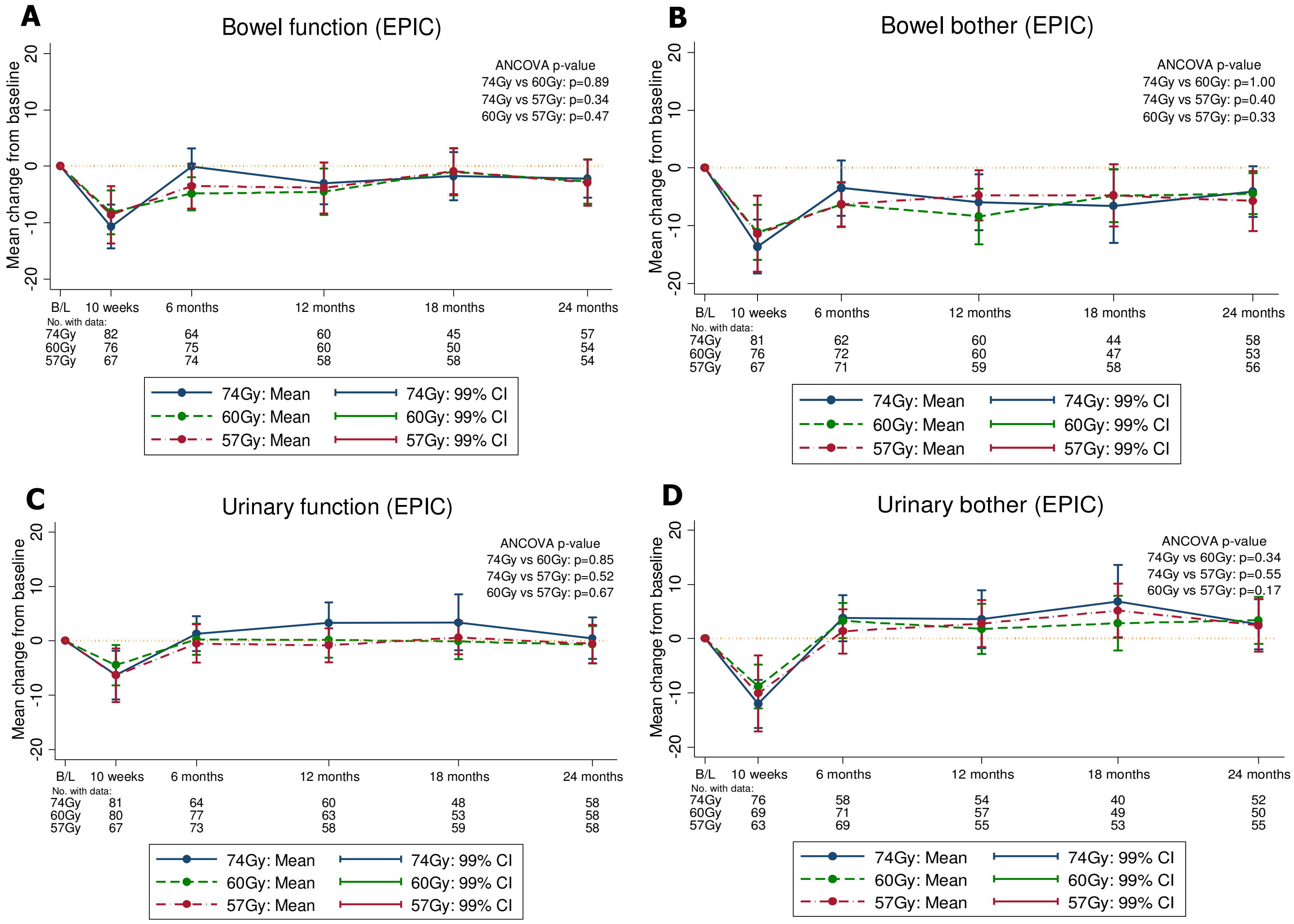

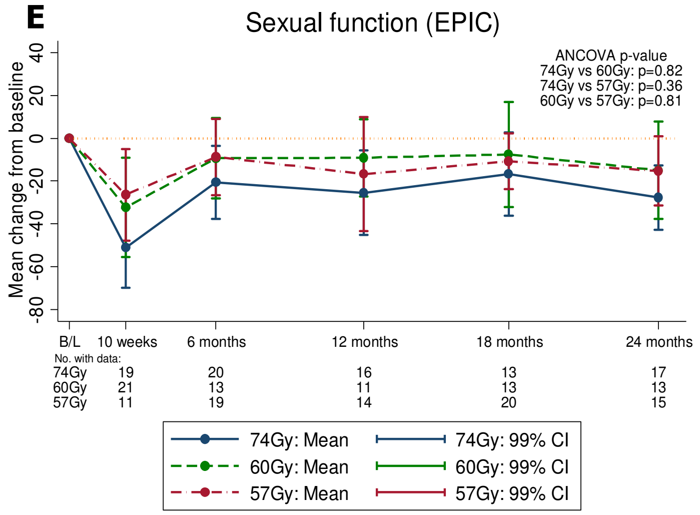

† For all urinary and bowel domain scores, to maximise numbers the pre-radiotherapy (PRT) score was used as a surrogate baseline score unless missing in which case the baseline score (BL) was used. Exact numbers for change from BL/PRT to 24 months are EPIC Bowel function PRT: 151, BL: 14, EPIC bowel bother PRT: 155, BL: 12 , EPIC Urinary function PRT: 162, BL: 12, EPIC Urinary bother PRT: 140, BL: 17.

Table 8a: Sensitivity analysis to check that the pre-radiotherapy (PRT) score was a valid surrogate baseline (BL) score for the "change from baseline" analysis.

**Overall bowel bother**				
Change in score	PRT/BL to 24 months		BL to 24 months	
	n	%	n	%
-4	1	0.09	0	0
-3	3	0.28	2	0.42
-2	29	2.75	10	2.11
-1	103	9.75	44	9.28
0	666	63.07	305	64.35
1	177	16.76	85	17.93
2	51	4.83	19	4.01
3	21	1.99	8	1.69
4	5	0.47	1	0.21
Comparison	PRT/BL to 24 months		BL to 24 months	
	OR (99% CI	p-value	OR (99% CI	p-value
60Gy vs 74Gy	0.85 (0.57-1.26)	0.29	1.25 (0.58-2.27)	0.34
57Gy vs 74Gy	0.84 (0.57-1.24)	0.25	1.05 (0.58-1.90)	0.58
60Gy vs 57Gy	0.99 (0.67-1.46)	0.95	0.86 (0.48-1.54)	0.51
**Overall urinary bother**				
Change in score	PRT/BL to 24 months		BL to 24 months	
	n	%	n	%
-4	2	0.19	1	0.21
-3	27	2.56	7	1.47
-2	62	5.89	20	4.2
-1	200	18.99	88	18.49
0	592	56.22	297	62.39
1	130	12.35	53	11.13
2	26	2.47	8	1.68
3	11	1.04	2	0.42
4	3	0.28	0	0
Comparison	PRT/BL to 24 months		BL to 24 months	
	OR (99% CI	p-value	OR (99% CI	p-value
60Gy vs 74Gy	1.30 (0.89-1.91)	0.07	0.81 (0.46-1.45)	0.36
57Gy vs 74Gy	0.99 (0.68-1.43)	0.92	0.74 (0.41-1.33)	0.19
60Gy vs 57Gy	0.76 (0.52-1.10)	0.06	0.92 (0.52-1.64)	0.70

Table 8b: Sensitivity analysis to check that the pre-radiotherapy (PRT) score was a valid surrogate baseline (BL) score for the "change from baseline" analysis.

The difference between baseline score at trial entry and the score pre-radiotherapy is compared below.

**Change from baseline score**	**-5**	**-4**	**-3**	**-2**	**-1**	**0**	**1**	**2**	**3**	**4**	**5**	**Total**
**Bowel symptoms**												
Overall bowel bother	0	0	1	9	66	563	70	16	9	2	0	736
Overall bowel bother (%)	*0*	*0*	*0.25*	*1*	*9*	*76*	*10*	*2*	*1*	*0.5*	*0*	
Rectal urgency	0	6	20	16	24	584	35	16	26	6	0	733
Rectal urgency (%)	*0*	*1*	*3*	*2*	*3*	*80*	*5*	*2*	*3*	*1*	*0*	
Faecal incontinence	0	0	2	0	3	89	2	2	0	0	0	98
Faecal incontinence (%)	*0*	*0*	*2*	*0*	*3*	*91*	*2*	*2*	*0*	*0*	*0*	
Rectal bleeding	0	-1	0	0	3	88	5	1	0	0	0	98
Rectal bleeding (%)	*0*	*1*	*0*	*0*	*3*	*89*	*5*	*1*	*0*	*0*	*0*	
Loose stools	0	3	3	12	136	448	107	12	7	0	0	728
Loose stools (%)	*0*	*0.5*	*0.5*	*1.5*	*19*	*62*	*15*	*2*	*1*	*0*	*0*	
Bowel frequency			0	0	2	92	3	0	0			97
Bowel frequency (%)			*0*	*0*	*2*	*95*	*3*	*0*	*0*			
Crampy pain	0	1	6	11	23	640	22	10	7	5	0	725
Crampy pain (%)	*0*	*0*	*1*	*2*	*3*	*88*	*3*	*1*	*1*	*1*	*0*	
Bowel distress		0	0	3	54	472	57	5	1	1		593
Bowel distress (%)		*0*	*0*	*0.5*	*9*	*80*	*10*	*1*	*0*	*0*		
**Urinary symptoms**												
Overall urinary bother	0	0	6	27	106	430	129	23	5	2	0	728
Overall urinary bother (%)	*0*	*0*	*1*	*4*	*15*	*59*	*18*	*3*	*1*	*0.5*	*0*	
Urinary control		0	0	1	60	588	89	2	1	0		741
Urinary control (%)		*0*	*0*	*0*	*8*	*79*	*12*	*0*	*0*	*0*		
Use of urinary pads			0	0	1	719	6	0	0			726
Use of urinary pads (%)			*0*	*0*	*0*	*99*	*1*	*0*	*0*			
Haematuria	0	1	0	0	0	93	0	1	0	0	0	95
Haematuria (%)	*0*	*1*	*0*	*0*	*0*	*98*	*0*	*1*	*0*	*0*	*0*	
Dysuria	0	0	0	1	3	84	4	1	1	0	0	94
Dysuria (%)	*0*	*0*	*0*	*1*	*3*	*89*	*4*	*1*	*1*	*0*	*0*	
**Sexual symptoms**												
Overall sexual bother	0	17	15	29	62	272	101	84	59	49	0	688
Overall sexual bother (%)	*0*	*2*	*2*	*4*	*9*	*40*	*15*	*12*	*9*	*7*	*0*	
Erection quality		0	2	5	20	260	136	136	131	0		690
Erection quality (%)		*0*	*0*	*1*	*3*	*38*	*20*	*20*	*19*	*0*		
Erection frequency	0	0	4	8	9	273	110	94	93	96	0	687
Erection frequency (%)	*0*	*0*	*1*	*1*	*1*	*40*	*16*	*14*	*14*	*14*	*0*	
Morning erections	0	0	0	3	13	220	185	86	44	11	0	562
Morning erections (%)	*0*	*0*	*0*	*1*	*2*	*39*	*33*	*15*	*8*	*2*	*0*	

Table 9: Sensitivity analysis to check validity of including patients who completed baseline QoL assessments having started endocrine treatment less than 1 month ago.

	BL QoL assessment before any endocrine treatment started		Endocrine treatment started less than one month before BL QoL assessment	
	N	%	N	%
**Quality of erections**				
Firm enough for intercourse	252	46.4%	337	46.2%
Firm enough for masturbation and foreplay only	114	21.0%	149	20.4%
Not firm enough for any sexual activity	85	15.7%	112	15.4%
None at all	77	14.2%	112	15.4%
Not available	15	2.8%	19	2.6%
Total	543	100.0%	729	100.0%
**Frequency of erections**				
Erection whenever desired	164	29.8%	219	29.6%
Erection >50% times desired	90	16.3%	122	16.5%
Erection 50% times desired	79	14.3%	96	13.0%
Erection <50% times desired	68	12.3%	94	12.7%
Never when desired	128	23.2%	181	24.5%
Not available	22	4.0%	28	3.8%
Total	551	100.0%	740	100.0%
**Morning erections**				
Very often	16	3.3%	20	3.1%
Often	69	14.1%	90	13.9%
Not often	91	18.6%	123	19.0%
Seldom	172	35.2%	219	33.8%
Never	132	27.0%	183	28.2%
Not available	8	1.6%	13	2.0%
Total	488	100.0%	648	100.0%
**Overall Sexual bother**				
No problem	230	41.7%	307	41.3%
Very small problem	75	13.6%	105	14.1%
Small problem	69	12.5%	96	12.9%
Moderate problem	78	14.1%	98	13.2%
Big problem	83	15.0%	113	15.2%
Not available	17	3.1%	25	3.4%
Total	552	100.0%	744	100.0%

Table 10: Numbers of patients per treatment group used pre-radiotherapy and at baseline for change from baseline to 24 months in bowel and urinary domains and overall bowel and urinary bother single items

EPIC bowel summary	74Gy	60Gy	57Gy	Total
Pre-radiotherapy	52	46	48	146
Baseline	4	5	5	14
**EPIC urinary summary**	**74Gy**	**60Gy**	**57Gy**	**Total**
Pre-radiotherapy	46	44	49	139
Baseline	5	6	5	16
**UCLA bowel function**	**74Gy**	**60Gy**	**57Gy**	**Total**
Pre-radiotherapy	251	239	259	749
Baseline	20	22	23	65
**UCLA urinary function**	**74Gy**	**60Gy**	**57Gy**	**Total**
Pre-radiotherapy	253	242	256	751
Baseline	19	20	25	64
**Overall bowel bother**	**74Gy**	**60Gy**	**57Gy**	**Total**
Pre-radiotherapy	325	312	334	971
Baseline	25	30	30	85
**Overall urinary bother**	**74Gy**	**60Gy**	**57Gy**	**Total**
Pre-radiotherapy	332	314	333	969
Baseline	26	27	31	84

The CHHIP QL centres. *Principal and main co-investigators according to centre (number of QL study patients recruited in bold).* † = CHHIP Trial Management Group member

Addenbrooke's Hospital, **79**, Dr Helen Patterson†, Dr Y Rimmer; Alexandra Hospital, Redditch, **15**, Dr Hamilton; Ayr Hospital, **25**, Dr R Mahmood, Dr J Ansari, Dr H Glen; Basingstoke and North Hampshire Hospital, **15**, Dr R Shaffer; Beatson West of Scotland Cancer Centre, **56**, Dr J Graham†, Dr M Russell, Dr J Wallace; Bedford Hospital, **39**, Dr R Thomas; Belfast City Hospital, **52**, Dr J O'Sullivan; Bradford Royal Infirmary, **7**, Dr A Henry; Bristol Haematology and Oncology Centre, **18**, Dr M Beresford, Dr A Bahl;Burnley General Hospital, **1**, Dr O Parikh; Charing Cross Hospital, **2**, Dr Mangar; Cheltenham General Hospital, **4**, Dr P Jenkins; Christie Hospital, Manchester, **92**, Dr J Logue, Dr A Choudhury†; Clatterbridge Centre for Oncology, **130**, Dr I Syndikus†; Clayton Hospital, Wakefield, **4**, Dr A Henry; Countess of Chester Hospital, **26**, Dr A Ibrahim; Eastbourne District General Hospital, **17**, Dr F Mckinna; Halton Hospital, **6**, Dr I Syndikus†; Hammersmith Hospital, **23**, Dr S Mangar; Heartlands Hospital, Birmingham, **3**, Dr A Zarkar; Hereford County Hospital, **9**, Dr A Cook; Ipswich Hospital, **86**, Dr C Scrase†; James Paget Hospital, Great Yarmouth, **4**, Dr R Wade; Kings Lynn and Wisbech NHS Hospital, **22**, Dr G Horan; Lincoln County Hospital, **64**, Dr M Panades; Maidstone District General Hospital, **2**, Dr Beesley; Mayday University Hospital, **10**, Dr R Huddart; Norfolk and Norwich University Hospital, **37**, Dr R Wade; North Wales Cancer Treatment Centre, **1**, Dr Al-Samarraie, Dr M Latif; Northampton General Hospital, **27**, Dr C Elwell; Poole General Hospital, **1**, Dr J Davies; Queen Elizabeth Hospital, Birmingham, **19**, Dr D Ford, Dr A Zarkar; Royal Blackburn Hospital, **1**, Dr O Parikh; Royal Bolton Hospital, **1**, Dr Elliot; Royal Marsden Hospital, London, **139**, Dr V Khoo†; Royal Marsden Hospital, Sutton, **294**, Professor D Dearnaley; Royal Oldham Hospital, **2**, Dr J Livsey; Royal Preston Hospital, **78**, Dr A Birtle; Royal Surrey County Hospital, **46**, Dr J Money-Kyrle; Royal Sussex County Hospital, **43**, Dr D Bloomfield; Royal United Hospital, Bath, **2**, Dr H Newman, Dr M Beresford; Southport General Infirmary, **59**, Dr C Eswar, Dr A Siva; St Bartholomew's Hospital, London, **14**, Dr P Wells; St James's University Hospital, Leeds, **15**, Dr C Coyle, Dr A Henry; Torbay District General Hospital, **1**, Dr Lydon; University Hospital Coventry, **34**, Dr A Stockdale; University Hospital of North Staffordshire, **20**, Dr F Adab; Velindre Hospital, Cardiff, **68**, Dr J Staffurth†; Warrington Hospital, **65**, Dr I Syndikus†; West Suffolk Hospital, **25**, Dr C Woodward; Western General Hospital, Edinburgh, **2**, Dr McLaren; Weston Park Hospital, Sheffield, **55**, Dr P Kirkbride, Dr C Ferguson; Whipps Cross University Hospital, London, **18**, Dr P Wells; Whiston Hospital, **68**, Dr Z Malik; Worcester Royal Infirmary, **10**, Dr A Stockdale; Worthing Hospital, **51**, Dr Bloomfield.

Conventional or Hypofractionated High Dose Intensity Modulated Radiotherapy for Prostate Cancer

Protocol Version

7.0

Protocol Number:

ICR-CTSU/2006/10007

CONFIDENTIAL: Information and data included in this protocol are privileged or confidential and are the property of ICR-CTSU. No person is authorised to make this public without the written permission of ICR-CTSU. These restrictions on disclosure will apply equally to all future information supplied to you, which is indicated as privileged or confidential. However, this material may be disclosed to and used by your staff and associates as necessary to conduct the clinical study.

MREC No.: 04/MRE02/10 **ISRCTN No.: ISRCTN97182923**

General correspondence to:

ICR-CTSU*
Section of Clinical Trials
Sir Richard Doll Building
Cotswold Road
Sutton, Surrey,
SM2 5NG, UK
Tel: +44 (0) 20 8722 4183
Fax: +44 (0) 20 8770 7876
Email:CHHIP-icrctsu@icr.ac.uk

***Institute of Cancer Research**
- Clinical Trials & Statistics Unit

Clinical correspondence to:

Professor David Dearnaley
Department of Academic Radiotherapy
The Royal Marsden Hospital
Downs Road
Sutton, Surrey, UK
SM2 5PT
Tel: +44 (0)20 8661 3271
Fax: +44 (0)20 8643 8809
Email: david.dearnaley@icr.ac.uk

This clinical trial protocol is intended to provide guidance and information only for the conduct of the CHHIP Trial in participating centres. It is not for use as a guide for the management of other patients outside of the trial.

If you have an urgent clinical query please contact:
Professor David Dearnaley on 020 8661 3271

The CHHIP trial has been scientifically approved by the Clinical Trials Awards and Advisory Committee (CTAAC) of Cancer Research UK and the Medical Research Council and is thus part of the NCRN/NCRI portfolio of prostate cancer trials.

CHHIP Trial - FINAL PROTOCOL VERSION 7.0: 19 October 2009

Approved by:

19 / 10 / 2009

Professor David Dearnaley
Chief Investigator

Date: 19/10/2009

INDEX

TRIAL SCHEMA 5
TRIAL SUMMARY 6
1. Background 7
1.1 Dose Escalation in Prostate Cancer 7
1.2 Hormone Treatment 8
1.3 Radiobiology of Prostate Cancer and Normal Tissue: Rationale for Hypofractionation 9
1.4 Rationale for Study Design 11
2. Objectives of the Study 13
2.1. To test the hypothesis that hypofractionated radiotherapy schedules for localised prostate cancer will improve the therapeutic ratio by either: 13
2.2. To limit acute and late gastro-intestinal and urological toxicity. 13
2.3. To evaluate different PSA related endpoints for local failure and distant metastases. 13
2.4. To extend the database of patients treated to escalated doses with dose volume histograms (DVHs) of normal tissues at risk and to relate these to common toxicity endpoints. 13
2.5. To develop a model to estimate normal tissue complication probability (NTCPs) of rectum and bladder for hypofractionated as well as conventional dose escalated radiotherapy schedules. 13
3. Trial Design 13
Figure 1 13
4. Patient Selection and Eligibility Criteria 14
5. Study Endpoints 15
5.1 Primary: 15
5.2 Secondary: 15
6. Randomisation, registration and treatment allocation 15
6.1 Registration & Randomisation 15
6.2 Allocation of treatment 15
7. Investigations and Assessment Procedures 16
7.1 Initial Assessment 16
7.2 During Hormone Therapy 17
7.3 During Radiotherapy 17
7.4 Post-radiotherapy treatment Follow-Up 17
7.5 Assessment of Disease Recurrence 17
7.6 Quality of Life Instruments 18
8. Treatment 19
8.1 Hormone Therapy 19
8.2 Radiotherapy Planning and Treatment 19
8.3 CT Planning for Radiotherapy 19
8.4 Target Volumes and Dose Assessment Points 20
Figure 2 22
Table 2 Summary of GTV, CTV and PTV Definitions and Dose Levels in Different Treatment Groups 23
Table 3 Outlining and Target Isodoses 24

Figure 3 25
8.5 Normal Tissue Contouring and Dose Volume Histograms 26
Table 4 Normal Tissue Dose Constraints 26
8.6 Radiotherapy Treatment Planning 27
8.7 Dose Constraints 28
8.8 Treatment Delivery 29
8.9 Verification and Accuracy 30
9. Adverse Events (AE) / Serious Adverse Events (SAE) 31
9.1 Definition of an Adverse Event 31
9.2 Definition of Serious Adverse Events 31
9.3 Reporting of Adverse Events 32
9.4 Expedited reporting of SAEs 32
9.5 Reporting related and unexpected SAEs 33
9.6 SAE follow up 33
10 Statistical Considerations 33
10.1 Part I: Two Centres 33
10.2 Part II: Multicentre 34
10.3 Part III: Multicentre 35
Table 5 Number required per arm 35
Table 6 Predicted % change in PSA control rate 36
11 Research Governance 37
11.1.1 Trial Administration and Logistics 37
11.1.2 Participating centres responsibilities 37
11.1.3 Quality Assurance of Radiotherapy Delivery 37
11.2 Investigator training 38
11.3 Case Report Forms 38
11.4 Protocol compliance/on site Monitoring 38
11.5 Trial Management 40
11.5.1 Trial Management Group 40
11.5.2 Trial Steering Committee 40
11.5.3 Data Monitoring Committee 40
11.6 End of Study 41
11.7 Archiving 41
11.8 Publishing policy 41
12 Confidentiality and Liability 41
12.1 Liability/Indemnity/Insurance 41
12.2 Patient Confidentiality 42
13 Ethical Considerations 42
14 Withdrawal of patients from study treatment 43
15 Financial Matters 44
16 Associated studies 44
16.1 Quality of Life (QOL) 44
16.2 Health Economics 46
16.3 Translational studies 46
17. References 48

TRIAL SCHEMA

CHHIP TRIAL

Initial Assessment

Biopsy-TURP/TRB
Blood test-PSA, FBC, Biochemistry
Local staging-rectal exam
Lymph node staging-MRI/CT
Metastases staging-Bone scan
Pre-treatment symptom and Quality of Life assessments[+#]

↓

Hormone Treatment

Total 3-6 months only *+

↓

Randomisation

↓

**Group 1**	**Group 2**	**Group 3**
74Gy/37#	60Gy/20#	57Gy/19#

↓

Pre-radiotherapy Assessment

Rectal exam **
Blood test
Symptoms and QoL assessments[#]

↓

Radiotherapy

Acute toxicity at Weeks 1-8, 10, 12 & 18
PSA at Weeks 10 & 18
Rectal exam** at Week 18

↓

Post-radiotherapy

- PSA- 6 mth to 5 yrs 6 monthly intervals then annually
- Late side effect (RTOG/LS), rectal exam** & QoL from 6mth to 2 yrs 6 monthly intervals, Yrs 2-5 annually. [#]
- Disease recurrence assessment

↓

End points

- Acute side effects
- Late side effects
- Biochemical (PSA) recurrence
- Distant recurrence
- Cause specific survival
- Overall survival

* May be omitted for patients with good risk disease (PSA≤ 10ng/ml <u>and</u> Gleason score ≤ 6 <u>and</u> T1c/T2a)

** Rectal examination may be omitted if the previous examination was normal <u>and</u> PSA ≤ 1.5ng/ml <u>and</u> no symptoms suggesting recurrence. [1]

\+ Patients who have already commenced hormonal therapy remain eligible but pre-hormone symptom scores and hormone measurement will be omitted

[#] The QoL sub-study has closed to patient recruitment.

TRIAL SUMMARY

Phase III randomised trials using conformal radiotherapy (CFRT) have shown that increasing radiation dose improves the control of localised prostate cancer and can be delivered safely without an increase in radiation related side effects. Recent studies on the radiobiology of prostate cancer have suggested that shorter courses of radiotherapy giving higher doses at each treatment (hypofractionated radiotherapy) may give improved cancer control for the same level of radiation related side effects. If this suggestion were to be confirmed, then treatments would become more convenient for patients for example 20 treatments over four weeks compared to 37 treatments over seven and a half weeks and radiotherapy resources would be better utilised. Intensity modulated radiotherapy (IMRT) techniques can now be designed which achieve a further improvement in conformality and normal tissue avoidance compared to CFRT. Suitable IMRT techniques will be used in this trial. The study will be undertaken in three stages. Part 1 is a randomised pilot study which will obtain preliminary data on side effects and has been undertaken in two centres (Royal Marsden Hospitals and Clatterbridge Cancer Centre); Part 2 has been expanded to include eleven centres and is powered to formally compare the side effects of the three treatment schedules; Part 3 has been approved as a national multi-centre study and will be powered to compare treatment efficacy. Part 1 of the study has been run by the Academic Unit of Radiotherapy at the RMH and is supported by the Unit's CR UK Programme Grant; Part 2 has been supported by the Dept of Health and Southern Prostate Cancer Collaborative to facilitate generalisibility of the hypofractionated and IMRT techniques. Part 3 is supported by CTAAC with quality assurance from the Dept of Health and NCRN.

1. Background

1.1 Dose Escalation in Prostate Cancer

Radiotherapy is one of the curative treatment options for localised prostate cancer [2, 3]. Considerable advances in radiation technology over the last decade have led to the development of conformal radiation treatments which more closely match the high dose volume to the tumour target whilst reducing the radiation to dose limiting normal tissues [4]. The potential advantages of these techniques is to enable a reduction in radiation related side effects as well as permitting the safe delivery of high doses of radiation which might improve treatment efficacy. Institutional experiences and results from phase I/II studies suggests that both these goals may be achievable [5-7] and that dose/response relationships exist for tumour control as well as dose/volume/complication relationships for the development of late normal tissue damage.

In a phase III randomised trial [8] we compared conventional and conformal radiotherapy (CFRT) at a standard dose of 64 Gy and showed a significant reduction in the dose limiting late side effect of proctitis using CFRT but no detriment in disease control. Three phase III trials using conformal photon beam treatment have reported gains in overall PSA control of between 6% and 12% using higher doses of radiation [9, 10, 72]. In the MD Anderson trial, which compared 70 and 78Gy, benefit (19% PSA control advantage) was restricted to men with a presenting PSA >10ng/ml [10]. The Royal Marsden Hospital (RMH) study compared 64 and 74Gy in combination with neoadjuvant androgen suppression [9]. The higher dose gave a 12% advantage in PSA control. Late morbidity was increased in high dose groups in both trials. In the recently reported Dutch multicentre trial in which 664 men were randomised to receive treatment with 78Gy or 68Gy, there was a 10% PSA control advantage for the higher dose, which was most clearly seen in men with intermediate risk disease (HR 0.6). Preliminary results using a proton beam boost (PROG 95-09) comparing doses of 70.2Gy equivalent and 79.2Gy equivalent suggests an 18% PSA control advantage in both low and intermediate risk groups [11]. These results build on the improvements in PSA control rates that have been previously reported in phase II studies in larger groups of men. [5-7, 12, 13]. For example, the Memorial Sloan Kettering Group have reported outcome from 1,100 men comparing doses in the range of 64 to 70Gy and 76 to 86Gy [14]. Using clinical stage, histological grade and

presenting PSA to define prognostic groups showed 5 year actuarial PSA control rates of 77% vs 90% (p= .05) for the most favourable group, 50% vs 70% (p= .001) for the intermediate group and 21% vs 47% (p= .002) for the unfavourable group treated to lower or higher doses respectively. A critical issue is whether or not PSA control will clearly relate to disease recurrence or to overall survival. A retrospective analysis from the Radiotherapy and Oncology Group (RTOG) suggests that dose escalation may indeed be related to improved survival. In their study, which included 1465 men treated in 4 protocols between 1975 and 1992, men with high grade cancers who received higher radiation doses (≥66Gy versus <66Gy) had a 20% lower risk of death from prostate cancer and a 27% reduction in overall mortality. This benefit was not seen in men with well or moderately differentiated cancers [15]. Over 3,000 men will be randomised in ongoing phase III studies of dose escalation in the UK (MRC RT01 trial), The Netherlands, France and N. America. The MRC RT01 trial completed recruitment of over 850 patients in December 2001 [51]. The remaining studies give doses of 68-73Gy in the control groups and 78-82Gy in the escalated dose group. As a result of the advantage demonstrated in the Royal Marsden/ICR trial and MD Anderson trials, 74Gy will become the standard dose for men treated in this study.

1.2 Hormone Treatment

An alternative strategy to improve the treatment results of radiation therapy is to use short or longer periods of adjuvant androgen suppression/blockade. Potential advantages of combined modality treatment include an additive or synergistic effect on tumour cell kill, a reduction in radiation target volume and reduction in the development of metastases. Phase III randomised trials have shown benefit for both short [16-19] and longer course hormonal therapy [20-23]. For short course (3-6 months) treatment long-term PSA control rates improve by 14-24% and with long term treatment (≥2 years) PSA control rates improved by 21%-31% with benefits in metastases-free, cause-specific and overall survival. All studies suggest improved outcome for long course treatment in men with locally advanced high grade cancer [23, 24]. Intermediate risk groups benefit from short course treatment but it is not yet certain if good prognosis patients benefit from adjuvant hormonal therapy in addition to high dose radiotherapy - NCRN approved EORTC Trial 22991 is addressing this question.

Several groups have measured the reduction in prostate and prostate target volume after initial hormone treatment which varied between 25-41% and showed a complementary increase in the sparing of rectum and bladder when initial hormone treatment was combined with CFRT [25-27].

1.3 Radiobiology of Prostate Cancer and Normal Tissue: Rationale for Hypofractionation

Recently there has been considerable discussion concerning the radiobiology of prostate cancer's response to irradiation [28-36]. In general, increased radiation fractionation provides an increasing therapeutic advantage between tumour control and late treatment related side effects, in that fractionation spares late responding normal tissues more than tumours because tumours normally respond as early responding tissue [37]. This sensitivity to change in fractionation is expressed mathematically in the linear-quadratic formalism and is quantified by the alpha-beta ratio [37]. In general, late responding normal tissues have a low alpha-beta ratio (usually taken as approximately 3 Gy) whereas early responding tissues responsible for acute radiation reactions and most cancers have a high alpha-beta ratio (usually 8-10 Gy). Fractionation spares tissues with a low alpha-beta ratio and radiotherapy schedules are designed so as to keep late radiation reactions at an acceptable level. For this reason, most cancers are treated with 1.8 - 2Gy daily fractions over a period of 6-8 weeks. However, studies deriving the alpha-beta ratio for prostate cancer from low dose rate brachytherapy treatments have suggested the alpha-beta ratio is 1.5 Gy (95% confidence intervals 0.8 - 2.2Gy) [38] and 1.49 Gy (95% confidence intervals 1.25 - 1.76) [29]. A further analysis using external beam radiotherapy with high dose brachytherapy estimated the alpha-beta ratio at 1.2Gy (95% confidence intervals .03-4.1Gy) [34]. If these estimates are accurate, they would predict that hypofractionated schedules for prostate cancer should produce tumour control and late treatment related sequelae that are at least as good or better than those currently achieved with currently standard schedules using 1.8-2.0Gy daily fractions. However, different assumptions in the models used for calculating the alpha-beta ratios can lead to estimates as high as 10Gy [28] and values of 8.5 Gy and 15.5 Gy have recently been derived by incorporating hypoxia into the modelling process [35].

Clinically hypofractionated external beam radiotherapy has been used for many years in the UK for a variety of malignancies, predominantly as a result of limited resources. In the past satisfactory results were claimed using a variety of hypofractionated treatment schedules for

prostate cancer varying from 50Gy in 20 fractions over 4 weeks [39], 50Gy in 16 fractions over 21 days [40] and 36Gy in 6 treatments over five weeks [41]. Many centres in the UK continue to use 4 week radiotherapy schedules using total doses of between 50 and 55Gy.

Contemporary reports of hypofractionated schedules are limited. A phase III trial in 936 men has compared 52.5Gy in 20 fractions with 66Gy in 33 fractions. Preliminary results appeared to show a 7% reduction in PSA control rate (49% vs. 56%) in the 20 fraction arm with hazard ratio for failure (short to long) of 1.20 (95% CI 1.0 to 1.44). Late toxicity was similar in the two arms (Grade 3/4 = 3%) [42]. A second, small, randomised controlled trial including 120 men compared a dose of 64Gy in 32 fractions with 55Gy in 20 fractions. After median follow up of 44 months, 4 year PSA control rates were similar (86.2% vs. 85.4% for hypo and standard fractionation respectively); there was a slight excess of rectal bleeding in the hypofractionated group [43]. Comparison of a large single institute series in which 705 men were treated to a dose of 50Gy in 16 fractions gave similar PSA control rates to schedules of 65-70Gy in 1.8-2.0Gy fractions with a low toxicity profile [44]. All of these studies are compatible with an α/β ratio for prostate cancer of ≤1.5-3.0Gy [45]. Additionally, a preliminary report from the USA [46] suggested that a dose of 70Gy in 2.5Gy fractions was at least as effective as 78Gy in 2Gy fractions. Presently there are no long-term data using higher dose hypofractionated radiotherapy. Phase I studies using 3Gy fractions have recruited in Manchester (57Gy, 60Gy) [47], Toronto (up to 66Gy) (personal communication) and Japan (69Gy) [48].

The alpha-beta ratio for late reactions in normal tissues is usually taken as 3Gy for skin, mucosa and bowel. However, human data is quite imprecise. For example, the alpha-beta ratio for late telangiectasia following breast irradiation is 2.8 (95% confidence intervals 1.7-3.8), for breast fibrosis the alpha-beta ratio has been reported as 1.9 (95% confidence intervals 0.8-3.0), and for bowel stricture and perforation following pelvic treatment the alpha-beta ratio is probably between 2.2 Gy and 8Gy. [37, 49]. However, a recent review suggests that the alpha-beta ratio for radiation induced proctitis may be relatively high at 5.4Gy (±1.5Gy) [50]. There is little information concerning the effect of overall treatment time on the development of late radiation reactions and using schedules that are only modestly shortened it may be that no overall time factor is required for either tumours or late complications [30]. The situation is also uncertain for acute reactions. Although the overall reduced dose used in hypofractionated

schedules would be expected to lower side effects if the overall treatment time was kept constant (e.g. by treating 5 x fortnight), decrease in overall time (treatment acceleration) might increase side effects.

If the radiobiological predictions of a low alpha-beta ratio for prostate cancer are correct, such shortened schedules may be associated with improvements in tumour control for a given level of radiation related side effects. If this is the case, then such schedules should become the standard approach to treatment as they would be more convenient for patients and make better use of radiotherapy resources. To date, no Phase III study of dose escalated conformal or intensity modulated radiotherapy using hypofractionated schedules has been performed.

1.4 Rationale for Study Design

We want to test whether there is an improvement in the therapeutic ratio using an hypofractionated radiotherapy schedule in prostate cancer. The study design is based on the biological hypothesis that the α/β ratio for prostate cancer is low (<3Gy). Two different strategies can be used to select appropriate dose levels in the hypofractionated groups. The first would be to assume a low alpha-beta ratio of 1.5Gy and then to calculate the iso effective dose required using any hypofractionated schedule. It would be predicted that both late and acute reactions would be reduced (assuming alpha-beta ratio of 3 Gy and 10 Gy respectively). The risk with this approach is that the tumour will be undertreated if the alpha-beta ratio were to be higher. The second strategy would be to aim for an iso-effective dose for late normal tissue complications assuming an alpha-beta ratio of 3 Gy. If the alpha-beta ratio for tumour (e.g. 1.5Gy) is lower than that for normal tissues (e.g. alpha-beta ratio = 3Gy) then if treatment groups are iso-effective for late normal tissue damage the hypofractionated schedule would have a higher tumour control probability. A difficulty arises, however, because of the imprecisely known alpha-beta ratios for both tumour and late responding normal tissues.

We have favoured a mixed strategy as this acknowledges the uncertainties in alpha-beta ratio for both prostate cancer and normal tissues. As 4 week schedules have been used in the UK and are familiar to clinicians, and some pilot data is already available, we have chosen to compare 4 week schedules with the standard 7.5 week treatment. A 3 group randomisation is preferable so that two points on the experimental hypofractionated schedule dose complication

response curve can be observed which should allow extrapolation to an iso-effective dose for tumour control compared to conventional 2Gy fractionation schedules. Table 1 shows the predicted 2Gy equivalent doses for a range of alpha-beta ratios comparing conventional 74Gy (2Gy fractions), high dose hypofractionated schedules, as well as previously studied "standard" hypofractionated treatments. The calculated 2Gy equivalent doses using a 20 fraction schedule are 61.1Gy and 58.0Gy for alpha/beta ratio of 3.0 and 1.5Gy respectively. Practically 3Gy fractionation schedules are attractive, and schedules of 60Gy in 20 fractions, and 57Gy in 19 fractions will be compared with standard treatment of 74Gy in 37 fractions; the hypofractionated schedules would be iso-effective for alpha beta ratios of 2.5Gy and 1.5Gy respectively. Assuming the alpha-beta ratio for normal tissues is 3Gy and the alpha-beta ratio 1.5Gy for prostate cancer, the hypofractionated group would show a therapeutic gain in that tumour control would improve for an iso-effect on normal tissues. If the alpha-beta ratio for prostate cancer is over 3Gy, however, there will be a relative dose detriment for tumour control for both groups and if the alpha-beta ratio for late reacting normal tissues were under 1.5Gy then there would be an increase in normal tissue complication rate for both groups. However, both hypofractionated groups are treated to a total dose in excess of what has been given using conventional rather than conformal radiotherapy techniques (50 - 55Gy in 20 fractions or 50Gy in 16 fractions) and it is anticipated that IMRT techniques will make a significant reduction on normal tissue complication rates. As above, if acute side effects were found to be dose limiting in the pilot study, overall treatment time could then be lengthened.

Table 1 Fractionation Schedules in Prostate Radiotherapy

Dose (Gy)	Dose/F (Gy)	No. F	2Gy Equivalent Dose α/β Ratio					
			1.0	1.5	2.0	3.0	5.0	10.0
50	3.13	16	69	66	63	60	58	54
55	2.75	20	68	66	65	64	61	58
57	3.0	19	76	74	71	68	65	62
60	3.0	20	80	78	75	72	68	66
70	2.5	28	82	80	78	77	75	73
70.2	1.8	39	66	66	66	68	68	69
77.4	1.8	43	72	73	74	74	76	78

2. Objectives of the Study

2.1. To test the hypothesis that hypofractionated radiotherapy schedules for localised prostate cancer will improve the therapeutic ratio by either:

Improving tumour control or
Reducing normal tissue side effects

2.2. To limit acute and late gastro-intestinal and urological toxicity.

2.3. To evaluate different PSA related endpoints for local failure and distant metastases.

2.4. To extend the database of patients treated to escalated doses with dose volume histograms (DVHs) of normal tissues at risk and to relate these to common toxicity endpoints.

2.5. To develop a model to estimate normal tissue complication probability (NTCPs) of rectum and bladder for hypofractionated as well as conventional dose escalated radiotherapy schedules.

3. Trial Design

Patients will be randomised between conventional radiotherapy fractionation using a total dose of 74 Gy in 37 fractions over 7.4 weeks using conformal and intensity modulated radiotherapy techniques and the experimental groups of 57Gy in 19 fractions over 3.8 weeks and 60Gy in 20 fractions over 4 weeks (Figure 1).

Figure 1

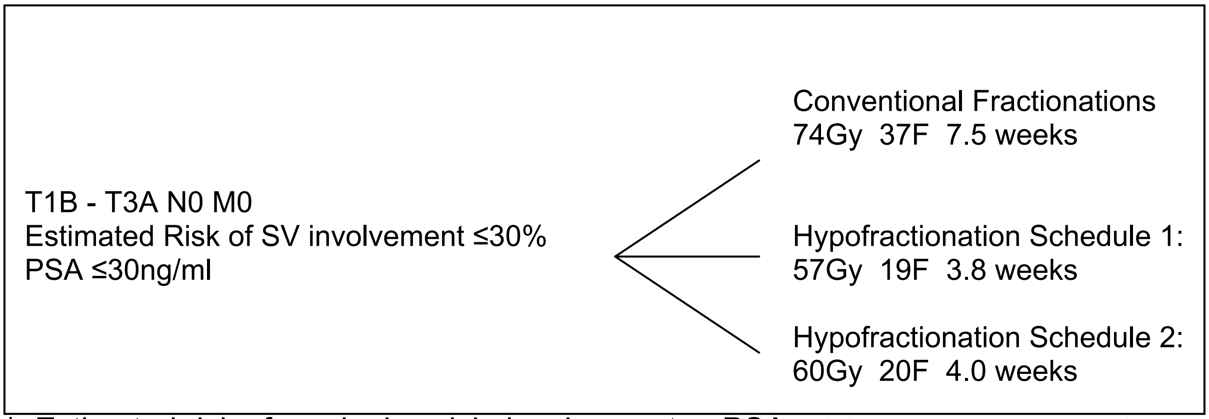

* Estimated risk of seminal vesicle involvement = PSA + ([Gleason score -6] x10) [52]

4. Patient Selection and Eligibility Criteria

Inclusion:

- Histologically confirmed, previously untreated locally confined adenocarcinoma of the prostate
- Clinical stage T1b – T3a, N0, M0. (1997 TNM system)
- PSA ≤ 30 ng/ml
- Estimated risk of seminal vesicle involvement* ≤ 30%
- WHO performance status 0 or 1
- Normal blood count (Hb >11g/dl, WBC > 4000/mm^3, platelets >100,000/mm^3)
- Written informed consent

Exclusion:

- Patients with T3 cancers with Gleason Sum ≥8 cancers are ineligible
- Prior pelvic radiotherapy or radical prostatectomy
- Previous androgen deprivation
- Life expectancy <10 years
- Previous active malignancy within the last five years other than basal cell carcinoma
- Co-morbid conditions likely to impact on the advisability of radical radiotherapy (e.g. previously inflammatory bowel disease, previous colorectal surgery, significant bladder instability or urinary incontinence)
- Bilateral Hip prostheses or fixation which would interfere with standard radiation beam configuration

* Estimated risk of seminal vesicle involvement[44]= PSA +([Gleason score-6] x10)(i.e. for the purposes of this study if Gleason score ≤6 then PSA must be ≤30ng/ml: if Gleason score=7 then PSA must be ≤20ng/ml: if Gleason score=8 then PSA must be ≤10ng/ml: if Gleason score= 9 or 10, patient is ineligible).

5. Study Endpoints

5.1 Primary*:*
Freedom from biochemical (PSA) failure or prostate cancer recurrence.
Biochemical failure defined according to Phoenix consensus guidelines[75] as Nadir + 2ng/ml.
The Nadir PSA level is the lowest value recorded at any time after commencement of hormone and/or radiotherapy treatment.

5.2 Secondary*:*

- Acute and late radiation induced side effects
- Development of metastases
- Recommencement of hormonal treatment for disease occurrence
- Cause specific and overall survival
- Aspects of quality of life and health economics
- Models of normal tissue and tumour control

6. Randomisation, registration and treatment allocation

6.1 Registration & Randomisation

Patients are initially registered to the trial after obtaining informed consent. Ideally, patients should be registered prior to commencing hormone therapy. To register a patient complete Registration forms 1 and 2 and fax to the CHHIP Trial Team on **020 8722 4368**. The patient will be allocated a unique registration number.

Patients should be randomised as close to the start of radiotherapy as possible. Patients are randomised by calling the ICR-CTSU. The patient's registration number will be required at the time of randomisation.

Patients are randomised by telephone through the ICR-CTSU

Tel: 020 8643 7150 (09.00 – 17.00 Monday to Friday)

The caller will be given the patient's unique trial identification number (Trial ID) and treatment allocation.

6.2 Allocation of treatment

Treatment allocation will be 1:1:1 and will use computer generated random permuted blocks. Randomisation will be stratified by treating centre and risk group (low, intermediate or high).
A letter confirming randomisation will be sent to the centre to confirm treatment allocation.

7. Investigations and Assessment Procedures

7.1 Initial Assessment

All patients are required to undergo the following pre-randomisation investigations:

- clinical history and physical examination
- histological evaluation of prostate biopsy to be assessed using the Gleason scoring system
- staging procedure:-
  - * local tumour staging - record clinical results of rectal exam and findings of TRUS or MRI (body or endorectal coil)
  - * lymph node staging - record findings of CT or MRI (body coil)
  - * metastases staging - bone scan (maybe omitted if PSA <u><</u>10ng/ml <u>and</u> Gleason Score <u><</u>7)
- full blood count and biochemistry to include creatinine, alkaline phosphatase, PSA, testosterone**, FSH, LH.
- symptoms** - bowel, urinary symptoms and potency will be recorded using RTOG, LENT-SOM, and Quality of Life instruments

** Patients who have already commenced hormonal therapy remain eligible but pre-hormone symptom scores and hormone measurement will be omitted

For Part III, patients will be stratified by risk group as defined by NCCN Practice Guidelines in oncology. [74]

Three groups of patients will be defined as:

- Low risk prostate cancer (Group L)
  ;clinical stages T1b/c or T2a, with PSA ≤10 and Gleason score ≤6

- Intermediate risk prostate cancer (Group I)
  ; presence of any of the following*: PSA 10-20, Gleason Score 7, clinical stage T2b

- High risk prostate cancer (Group H)
  ;presence of any of the following: PSA >20, Gleason Score 8-10, clinical stage T3a

; TNM 1997 Classification

* Excluding patients with any high-risk feature

7.2 During Hormone Therapy

- PSA to be measured at 6 weeks and 12 weeks (prior to commencement of radiotherapy)
- Bowel, urinary symptoms and potency will be assessed using RTOG and LENT-SOM
- Rectal examination* prior to radiotherapy

7.3 During Radiotherapy

• Acute toxicity assessments (RTOG)	Weeks 1-8, 10, 12 and 18.
• PSA	Weeks 10 and 18
• Rectal examination*	Week 18

7.4 Post-radiotherapy treatment Follow-Up

- PSA - 6 months (26 weeks after commencement of RT), 12 months, 18 months, 24 months, Years 2-5: 6 monthly intervals; Years 6-15: annual intervals
- Hormones - 12 months after commencement of RT
- Late side effect assessment (using RTOG and LENT-SOM) - 6 months (26 weeks after commencement of RT), 12 months, 18 months, 24 months; Years 2-5: annual intervals
- Rectal examination* - 6 months (26 weeks after commencement of RT), 12 months, 18 months, 24 months; Years 2-5: annual intervals
- Health Resource questionnaire - 6 months (26 weeks after commencement of RT), 12 months, 18 months, 24 months; Years 2-5: annual intervals

*Rectal examination may be omitted if the previous examination was normal and PSA≤1.5ng/ml and no symptoms suggesting recurrence. [1]

7.5 Assessment of Disease Recurrence

Full assessment of disease will be undertaken if there is significant clinical or biochemical evidence of disease recurrence that will include CT or MR of the pelvis and bone scan. There should be clinical evidence of recurrence or PSA levels should be: (a) at least 10ngs/ml and (b) >50% of presenting PSA level to trigger re-evaluation [53] unless (c) the PSA doubling time is ≤6 months or high grade (Gleason 8-10) disease was initially present and PSA >5ngs/ml. Alternatively, reassessment should be undertaken with lower PSA levels if the decision is made to recommence hormone therapy.

7.6 Quality of Life Instruments

This sub-study has now closed to recruitment.

The instruments used will be the FACT-P (Prostate) and UCLA/RAND Prostate Cancer Index. However, the UCLA/RAND Prostate Cancer Index will be replaced with the updated version. The other instruments to be used will be the combined versions of the Expanded Prostate Cancer Index Composite (EPIC) plus SF-12 questionnaires. The Quality of Life sub-study will be discussed further in section 16.

It is essential to explain to the patient that the QL questionnaire is an important part of their assessment in the trial, and that all sections and questions should be answered even if the patient feels them to be irrelevant.

8. Treatment

8.1 Hormone Therapy

Androgen deprivation will be achieved using LHRH agonists in conjunction with initial cyproterone acetate* (CPA) to prevent 'flare' phenomenon. CPA may commence on or during the week before the day of the first LHRH agonist injection and should be given for at least two weeks after the LHRHa injection. Monthly depot injections of LHRH analogues should be used as 3 monthly depot preparations have a prolonged median duration of action. The duration of androgen deprivation should be at least three months (maximum six months) prior to commencement of radiotherapy and should continue until the end of radiotherapy treatment. The last monthly depot injection should be given within 1 week of the start or during radiotherapy. Alternatively bicalutamide 150mg daily may be given and should continue for 2 months[+] after the end of radiotherapy. Hormone treatment may be omitted for patients with good risk disease (T1c/T2a and Gleason score ≤6 and PSA ≤10ng/ml).

*Equivalent alternatives are permissible.

[+]This aims to mimic the duration of action of monthly LHRHa depot preparations. If troublesome gynaecomastia or breast pain develops, Tamoxifen 10mgs once or twice weekly may be given.

8.2 Radiotherapy Planning and Treatment

Following randomisation patients will be allocated to one of the three treatment groups: planning methods and treatment delivery and verification will be specified by each participating centre and will be the same for each group. Radiotherapy treatment should start after a minimum of 3 months and maximum of 6 months of hormonal treatment. Patients with a single prosthetic hip may be included in the trial. Beam angles for such patients should be chosen carefully to avoid having treatment fields entering through the prosthesis. Any significant image artifacts ("streaking" and/or "shadowing") should have their densities over-ridden to that of water.

8.3 CT Planning for Radiotherapy

Patients will have planning CT scans after at least two months hormone therapy prior to commencement of radiotherapy. Prostate and planning target volumes will be defined on CT

scans which will be taken at ≤ 5 mm intervals (≤ 5mm slice thickness). The bladder will be comfortably full, (patients to drink approximately 350 ml during the hour pre scan) and the rectum should ideally be empty of both faeces and flatus; the routine use of micro enemas (e.g. relaxit) is permissible. Positioning/immobilisation will be using approved departmental methods as specified in 7.8 for treatment delivery. Scans will be taken from the bottom of the sacro-iliac joints to the penile urethra (usually 1 cm below ischial tuberosities will be adequate).

8.4 Target Volumes and Dose Assessment Points

Volumes will be defined according to 1993 ICRU report 50 and supplement report ICRN 62: Prescribing, Recording and Reporting Photon Beam Therapy.

Two groups of patients will be defined:

1) Group 1 Low risk of seminal vesicle involvement
   a) clinical stages T1b/c or T2a/b and with PSA + ((Gleasons score -6) x10) <15

2) Group 2 Moderate or high risk of seminal vesicle involvement
   a) clinical stages T1b/c or T2a/b, and with PSA + ((Gleasons score -6) x10) >15
   b) T2c or T3a

GTV is prostate only for both Groups 1 and 2.
CTV1 is prostate and base of seminal vesicles (proximal 2cm) with 5mm margin for Group 1
CTV1 is prostate and seminal vesicles with 5mm margin for Group 2
CTV2 is prostate only for Groups 1 and 2 with 5mm margin
CTV3 is prostate only for Groups 1 and 2

The PTV 1-3 adds a 5mm margin to the relevant CTV, except that for PTV 2/3 there will be 0mm margin posteriorly or posterior inferiorly (i.e. towards the rectum).

<u>Outlining of Target Volumes</u>

In practice, PTV1 is constructed by growing a 1cm isotropic margin around the outlined prostate and all or part of the seminal vesicles. Clinical judgement should be used to ensure that inappropriately large volumes of rectum or bowel are not included in the target volume if

the seminal vesicles wrap around the rectum or small bowel or sigmoid colon are present within the target volume. For PTV2 a uniform margin of 1cm is added to the prostate alone except towards the rectum where a 5mm margin is used. Exceptionally, if there is a suspicion but not certainty - in this case the patient would not be eligible for the trial - of seminal vesicle involvement on MR scan the base of seminal vesicles can be included in PTV2. Target volumes, outlining and target isodoses are summarised in Tables 2 and 3 for low and moderate risk groups. The dose distribution to be obtained can be regarded as a core high dose region (PTV3) and two surrounding shells PTV2 - PTV3 and PTV1 - PTV2 (Figure 3). Target isodoses have been designed to achieve the following aims (Table 2 + 3):

1. Minimum (defined as to 99% of the target volume) to PTV1 will be the equivalent of 54Gy in 2Gy fractions prescribed to the isocentre and achieving target coverage by 95% isodose. This will be achieved having a 76% minimum isodose coverage. (To achieve the equivalent of 54Gy in 2Gy fractions for the conventional fractionation group, and assuming an alpha-beta ratio equal to 3, implies treating to 59.2Gy in 1.6Gy fractions. Minimum isodose coverage is therefore 100 x 59.2/74 x 0.95 = 76%).
2. Median dose to the outer shell (PTV1 - PTV2) will be the equivalent of 100% of 54Gy equivalent and equates to the 80% isodose.
3. Minimum dose to PTV2 will be the equivalent of 70Gy in 2Gy fractions prescribed to the isocentre . This will be achieved by having a 91% minimum isodose coverage. Median dose to the inner shell (PTV2 - PTV3) will be the equivalent of 70Gy and equates to the 96% isodose.
4. Median dose to PTV3 will be the equivalent to 74Gy (i.e. 100% ± 1%) with a minimum 95% isodose coverage.

Figure 2

Grade of distension	Illustration	Description
None	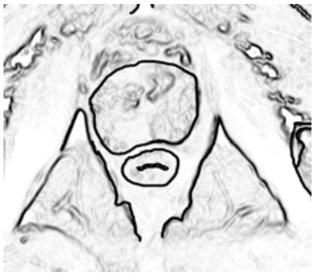	• Rectal walls opposed
Minimal	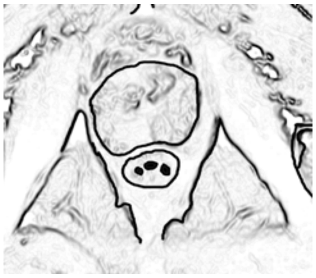	• Minimal rectal air
Small	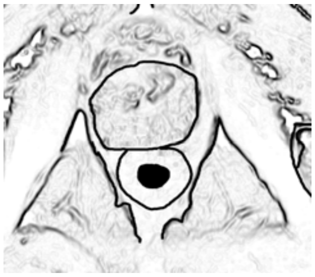	• No prostate distortion.<br>• Convex posterior prostatic border<br>• < 30% contact of rectum on posterior prostate border
Moderate	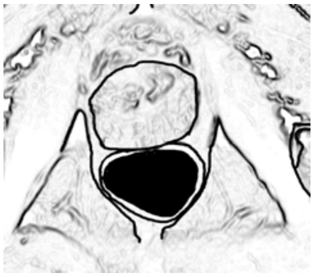	• Posterior prostatic border flattened<br>• 30-80% contact of rectum on posterior prostate border
Large	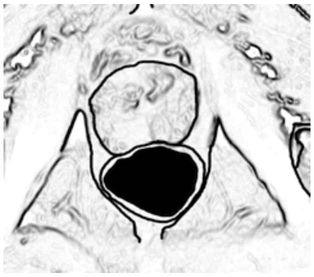	• Concave posterior prostatic border<br>• > 80% contact of rectum on posterior prostate border

Subjective grading of rectal distension

Padhani 1999 IJROBP [54]

Table 2 Summary of GTV, CTV and PTV Definitions and Dose Levels in Different Treatment Groups

Please use table 3 for outlining instructions

Low Risk Group		Moderate Risk Group		Dose				Minimum Isodose Coverage
				74 Gy Group	60 Gy Group	57 Gy Group	2 Gy equivalent[+++]	
GTV1	P	GTV1	P	59.2	48	45.6	54 Gy	76%
CTV1	P+base of SV+5mm	CTV1	P+SV+5mm					
PTV1	CTV +5mm	PTV1	CTV +5mm					
GTV2	P	GTV2	P	71	57.6	54.7	70 Gy	91%
CTV2	P+5mm	CTV2	P± base of SV[++] +5mm					
PTV2	CTV +5mm/0mm*	PTV2	CTV + 5mm/0mm*					
GTV3	P	GTV3	P	74	60	57	74 Gy	95%
CTV3	P+0mm	CTV3	P+0mm					
PTV3	CTV +5mm/0mm[+]	PTV3	CTV + 5mm/0mm[+]					

* 0mm posteriorly toward rectum unless moderate to large rectum (see diagram) then 5mm posteriorly towards rectum to be individualised for each CT image

[+] 0mm posteriorly towards rectum all patients

[++] Include base if T3B on MRI

[+++] Calculated for $\alpha/\beta = 3.0$ for 74Gy Group

Table 3 Outlining and Target Isodoses

	Low Risk	Moderate Risk	Target Isodose
PTV1	Outline: Prostate + base (proximal 2cm) of SV<br>Add: 10mm margin all directions	Outline: Prostate + SV<br>Add: 10mm margin all directions	76% minimum<br>≥ 80% median to PTV1 - PTV2
PTV2	Outline: Prostate only<br>Add:* 10mm margin except 5mm towards rectum unless moderate/large degree of rectal distension when 10mm towards rectum (only use 10mm on individual CT slices showing moderate/large rectal distension)		≥ 96% median to PV2 - PTV3
PTV3	Outline: Prostate only<br>Add: 5mm margin except 0mm towards rectum (all cases)		100%(±1%) median<br>95% minimum

* Note: PTV2 can be generated by adding a uniform margin to PTV3

Figure 3

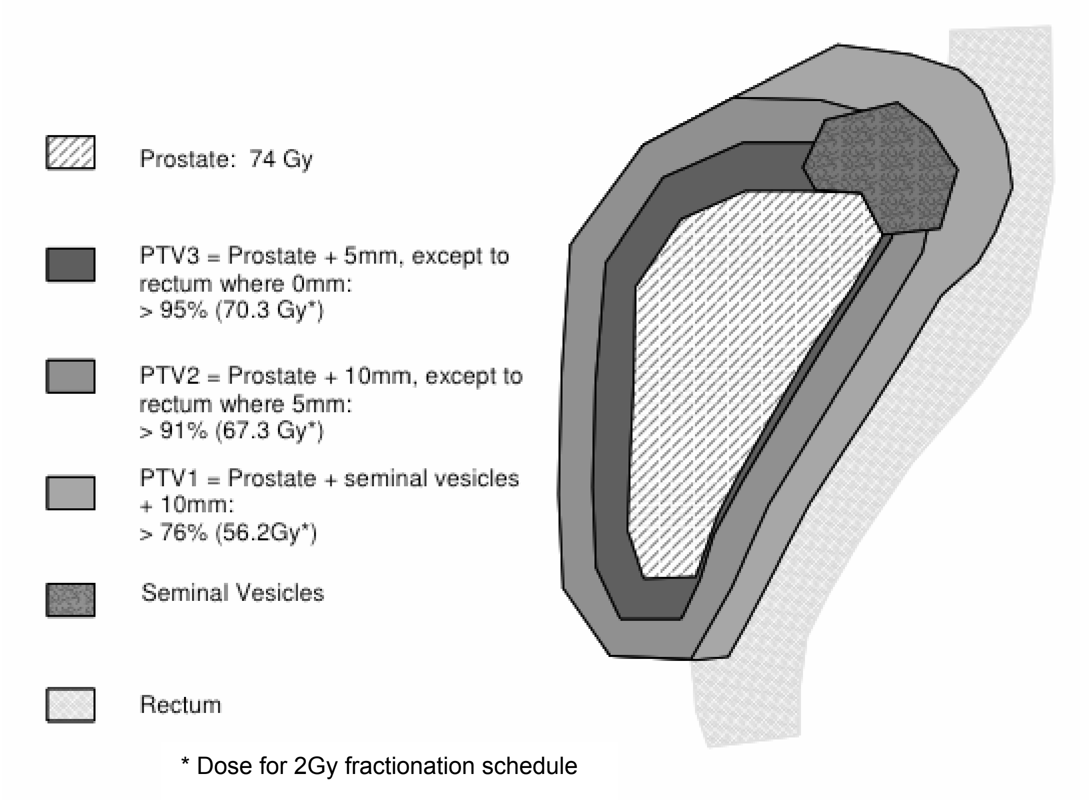

Prostate: 74 Gy
PTV3 = Prostate + 5mm, except to rectum where 0mm: > 95% (70.3 Gy*)
PTV2 = Prostate + 10mm, except to rectum where 5mm: > 91% (67.3 Gy*)
PTV1 = Prostate + seminal vesicles + 10mm: > 76% (56.2Gy*)
Seminal Vesicles
Rectum
* Dose for 2Gy fractionation schedule

8.5 Normal Tissue Contouring and Dose Volume Histograms

Normal tissues outlined will include bladder, rectum, femoral heads and skin. The normal tissues will be outlined as solid organs by defining the outer wall of rectum, bladder and bowel. Bladder should be outlined from base to dome. The rectum should be outlined from the anus (usually at the level of the ischial tuberosities or 1cm below the lower margin of the PTV whichever is more inferior) to the recto-sigmoid junction. The recto-sigmoid junction can usually be identified on the CT slice where the bowel turns anteriorly and to the left. This will give a length of 10-12cm in most cases. Any additional bowel in the volume should be outlined separately.

Whenever possible dose volume histogram data evaluating dose to the GTV, PTV and organs at risk (rectum, bladder, femoral heads, bowel, urethral bulb) will be collected.

A synopsis of dose volume histogram data will be collected prospectively in all patients (see Table 4) and whenever possible dose cube data for the entire distribution will be stored for subsequent more detailed analysis.

Table 4 Normal Tissue Dose Constraints

	Dose for 2Gy/# Prescribed Dose	Dose (%)	Max Vol (% or cc)
Rectum	30	41	80%
	40	54	70%
	50	68	60%
	60	81	50%
	65	88	30%
	70	95	15%
	74	100	3%
Bladder	50	68	50%
	60	81	25%
	74	100	5%
Femoral Heads	50	68	50%

Bowel	50	68	17cc
Urethral Bulb	50<br>60	68<br>81	50%<br>90%

8.6 Radiotherapy Treatment Planning

Forward or inverse 3D planning will be undertaken using standard beam arrangements to achieve the required dose distributions in a single treatment 'phase'. These may include 3 or 4 fields (anterior/lateral/posterior) or 5 fields or more if inverse planning is utilised. The beam arrangements used in any centre must be identical for the different treatment groups and must be approved by the Radiotherapy Quality Assurance Group. Dose conformation may be achieved either using static multiphase shielding using a multileaf collimator or alternatively “step and shoot” or “moving leaf” intensity modulated dose distributions may be generated. Tissue inhomogeneity corrections will be made for the femoral heads either on a pixel by pixel or using a standardised value of bone density.

Three dimensional dose distributions should be produced. The dose distribution should be assessed for coverage of the PTV and normal tissues using appropriate transverse sagital and coronal views.

The CHHIP physics plan assessment form must be completed and assessed against the dose volume constraints (Table 4), approved by clinician and sent immediately to the ICR-CTSU, either electronically (CHHIP-icrctsu@icr.ac.uk) or by fax on 020 8722 4368.

The plan including CT images, structures, plan and dose cube, should be exported and sent on CD to the CHHIP QA Physicist.

8.7 Dose Constraints

ICRU guidelines for IMRT treatments have not yet been designed. In this study median dose to target volumes will be described and minimum and maximum doses to target volumes will be defined by isodoses which include >99% and <1% of target volumes respectively. Minimum and maximum doses (to 99% and 1% of the volume respectively) within the PTV would normally be ≥95% and ≤105% respectively. Hot spot dose outside the PTV will not exceed 105%. Dose to organs at risk outside the PTV will not exceed the median prescribed dose to PTV3. The dose to 50% of the femoral heads should not exceed 68% of the prescribed dose and the maximum dose (to 1% of the volume) should not exceed 75% of this dose. Dose constraints for rectum [10, 55-62] bladder [58, 63], femoral heads [64] urethral bulb have been derived from the literature found on data from the MRC RT01 Trial and RTOG studies [65-66] to produce low and acceptable grade 1 to 3 complications. (Table 4). For the rectum dose constraints for 50Gy to 74Gy must be attained (see below), dose constraints at 30Gy and 40Gy are for guidance only (and found on some preliminary data from MRC RT01) as are the limits for the urethral bulb.

If individual plans fail to meet the constraints, target volumes and dose distributions will be reviewed to produce a clinically acceptable option. In general, median doses to PTV3, and surrounding target shells (PTV2 minus PTV3 and PTV1 minus PTV2) should be maintained when possible with some compromise to minimum target dose coverage. Inverse planning is encouraged if the initial dose distribution was produced using a 'forward planned' IMRT solution.

Rules to be followed if dose constraints are not met.

1) Rectum.

If more than one of the rectal dose constraints (excluding 30Gy and 40Gy ‘guidance levels) is "missed" the plan should be reviewed and the following steps taken to ensure that the plan comes back within tolerance (i.e. at least 4 or 5 of the constraints are met):

(a) review the target volume, ensure that PTV3 does not overlap the rectum at all and that, in the sagittal plane, PTV2 only overlaps the rectum by 5mm. Modify the target volume if the seminal vesicles wrap around the rectum (e.g. include only proximal 1-2cm)

(b) reduce the margin in the direction of the rectum. PTV1 may be reduced from 10mm to 7mm and PTV2 from 5mm to 2mm.

(c) If the rectal dose constraints are now not adequately achieved, dose should be reduced by up to 11% (i.e. a reduction of up to 4x2Gy fractions or 2x3Gy fractions). The dose constraints for all limiting organs should then be recalculated using the original intended dose as "100%". This will mean that the various prostate target volumes are recorded as being "under dosed".

If after these three manoeuvres the patient remains out of tolerance, the patient should be withdrawn from the trial and managed according to clinical judgement.

2) If bladder constraints are out of tolerance this is likely to be due to poor bladder filling in all cases and appropriate patient instruction should solve the problem, although ideally the patient should be re-scanned with a more completely filled bladder.

3) If femoral head tolerance is exceeded a new plan should be prepared

4) Bowel tolerance level should not be exceeded. This should be a very uncommon event and only occur if a loop of bowel has become fixed in the lower pelvis.

5) Urethral bulb constraints are for guidance only – the lower the dose the more likely potency will be retained but dose to the prostate area should not be compromised.

8.8 Treatment Delivery

All fields will be treated daily on a linear accelerator of ≥ 5MV. The planned overall treatment times will be 7.5 weeks for patients receiving 74Gy and 3.8 or 4 weeks for those receiving 57 and 60Gy respectively. A maximum delay of 5 treatment days may be permitted during therapy to allow for technical difficulties. If for technical reasons a

delay for longer than this period is likely, a maximum of 5 treatments may be given with unshaped fields to the patient group receiving 74Gy and 3 such treatments in patients receiving hypofractionated schedules. If patients, particularly in the hypofractionated groups, develop significant (Grade ≥2) acute toxicities, treatment 'gaps' may be introduced to allow side effects to settle before continuing therapy.

For the hypofractionated treatment schedules overall time of treatment should be at least 27 days (for the 20 fraction schedule) and 26 days for the 19 fraction schedule. This is to avoid undue shortening of overall treatment time and, in practice, means that these patients should start treatment on a Wednesday to Friday.

Patient immobilisation and treatment accuracy will be achieved by the placing of anterior and lateral tattoos at the level of the symphysis pubis, laser alignment during treatment set-up and positioning of the legs and feet using footstocks. The Radiotherapy Quality Assurance Group will approve and monitor each centre's procedures.

8.9 Verification and Accuracy

Appropriate dose verification will be performed before treatment if IMRT inverse planning is utilised. Beam calibrations should be performed according to a specified protocol preferably that described in the IPSM report [67]. Beam calibration will be assessed using methods defined by the Quality Assurance Group.

Suitable simulator films or digitally reconstructed radiographs (DRR) will be obtained to verify the orientation and alignment of the isocentre on the linear accelerator. Port films or images will be taken so that beam alignment and configuration can be confirmed. Orthogonal anterior and lateral images will be taken to assess the position of the isocentre in relationship to simulator films or DRR.

At least 3 portal images will be taken during week 1 and subsequently at weekly intervals or as additionally appropriate if patient positioning is problematic.

Port images will be compared to simulator images or digitally reconstructed images from CT. Treatment accuracy to within 3mm is to be obtained whenever possible and positioning errors ≥5mm are unacceptable. Corrections of patient positioning and

appropriate resimulation will be employed if errors greater than this magnitude are apparent before the next radiotherapy fraction is delivered. The Radiotherapy Quality Assurance Group will approve and monitor each centre's procedures.

9. Adverse Events (AE) / Serious Adverse Events (SAE)

9.1 Definition of an Adverse Event

An 'adverse event' is any untoward medical occurrence in a patient administered a research procedure; where the events do not necessarily have a causal relationship with the procedure.

For the purpose of this trial, any detrimental change in the patient's condition subsequent to the start of the trial (i.e. randomisation) and during the follow-up period, which is not unequivocally due to progression of disease (prostate cancer), should be considered as an AE.

Whenever one or more signs and/or symptoms correspond to a disease or well-defined syndrome only the main disease/syndrome should be reported. For each sign/symptom the highest grade observed since the last visit should be reported.

9.2 Definition of Serious Adverse Events

A serious adverse event is any untoward occurrence, that:

- results in death
- is life-threatening
- requires hospitalisation or prolongation of existing hospitalisation
- results in persistent or significant disability or incapacity or
- consists of a congenital anomaly or birth defect
- additionally RTOG Grade≥4 acute or late radiation side effects i.e. related to study treatment, will be regarded as an SAE

A related adverse event is one which has been assessed by the Principal Investigator and/or Chief Investigator (or nominated representative), as resulting from any of the trial treatments or procedures.

An unexpected adverse event is any type of event not listed in the protocol as an expected occurrence.

9.3 Reporting of Adverse Events

Adverse events will be collected from the time of randomisation to the end of the follow-up period. Adverse events should be recorded in the appropriate section of the CRF.

Due acknowledgement has to be given to likely co-morbidity and co-morbid events in an elderly and ageing male population, many of whom will die from diseases unrelated to prostate cancer and its treatment.

The following are possible anticipated treatment related AEs/SAE's (i.e. expected occurrences) which are not subject to expedited reporting but all such serious events should be reported in the appropriate section of the CRF.

Bone fractures
Bowel strictures
Second Malignancies
Ureteric obstruction

9.4 Expedited reporting of SAEs

All SAEs occurring within 30 days of study procedures being administered and not listed above, are subject to expedited reporting. In addition RTOG grades ≥4 acute or late radiation side effects occurring within 5 years of radiotherapy treatment are subject to expedited reporting.

All SAEs must be reported within 24 hours using the CHHiP SAE form. The form must be sent by FAX to The Institute of Cancer Research – Clinical Trials and Statistics Unit (ICR-CTSU) on **020 8722 4368**. It must be completed, signed and dated by the Principal Investigator or nominated representative.

ICR-CTSU will send the SAE to the Chief Investigator (or nominated representative) for review of causality and expectedness.

9.5 Reporting related and unexpected SAEs

If an SAE is assessed as related and unexpected ICR-CTSU will report this to the main REC within 15 days from the date the ICR-CTSU became aware of the event.

9.6 SAE follow up

For each SAE, the subject must be followed-up until clinical recovery is complete and laboratory tests have returned to normal, or until the condition has stabilised. Information on final diagnosis and outcome of SAEs which may not be available at the time the SAE is initially reported should be forwarded to the ICR-CTSU in the timeframe requested.

10 Statistical Considerations

10.1 Part I: Two Centres

Design:

Standard arm	74 Gy in 37 fractions
Experimental arm A	60 Gy in 20 fractions
Experimental arm B	57 Gy in 19 fractions

Randomised on a 1:1:1 basis.

Sample size:

The sample size for this study will be determined by the rate of ≥ grade 2 symptoms reported at 2 years. Previous studies have found that the rate of ≥ grade 2 long term complications is around 15% with an upper limit of the confidence interval as high as 25%.

For each of the arms in this randomised phase II study we would like to rule out this upper limit of 25% (p_0 = 75%). We expect the rate of ≥ grade 2 complications to be

better than the previous studies, around 10% (p_1 = 90%), for the standard arm because of the IMRT radiotherapy technique used.

Using the Simon single stage design (using exact p-values [68]) with power of 87.8% and a one sided alpha of 0.045 we will recruit 50 patients per arm. In each arm if 8 or more patients develop ≥ grade 2 complications at 2 years the study arm will be rejected. This ensures that the 25% upper limit of the complication rate at 2 years is ruled out. This design allows for patient drop out during the course of the study. Each study arm will be allowed 5 patients to drop out and still have 84.1% power to detect these effects. If only 45 patients are evaluable at 2 years then 7 or more patients with ≥ grade 2 complications would result in rejection of the treatment.

There will therefore be a total of 150 patients recruited to this study.

10.2 Part II: Multicentre

The aim of the second stage of the study is to rule out a maximum toxicity in the experimental arms twice that in the standard arm.

Therefore, for each of the experimental hypofractionated arms in this randomised phase II study we would like to rule out this upper limit of 20% (p_0 = 80%). We expect the rate of ≥ grade 2 complications to be better than the previous studies, around 10% (p_1 = 90%).

Using the Simon single stage design (using exact p-values [68]) with power of 95.6% and a one sided alpha of 0.037 we will recruit 150 patients per arm. In each arm if 22 or more patients develop ≥ grade 2 complications at 2 years the study arm will be rejected. This ensures that the 20% upper limit of the complication rate at 2 years is ruled out.

This design allows for patient drop out during the course of the study. Each study arm will be allowed 15 patients to drop out and still have 95.2% power to detect these effects. If only 135 patients are evaluable at 2 years then 20 or more patients with ≥grade 2 complications would result in rejection of the treatment.

There will therefore be a total of 450 patients recruited to this part of the study.

It may also be possible to detect differences in the biochemical control rate in this part of the study. With 150 patients per arm we could detect a 16% difference in biochemical control (70% vs 54%) for control vs experimental arm respectively with 81% power using a 2 sided alpha of 0.05. This will allow us to rule out treatments that are clearly inferior to the standard arm. Depending on the alpha/beta ratios we would only expect this in the 57 Gy arm if the alpha/beta ratio for prostate cancer is >5Gy (see Table 5).

10.3 Part III: Multicentre

The third stage of this trial will be a multicentre national trial (721 patients per arm). The aim of Part III of the CHHIP trial is to demonstrate non-inferiority between the experimental arms and the control arm. The biochemical PSA control rate in the standard arm is assumed to be 70% at 5 years. Table 5 gives the number of patients required to demonstrate non-inferiority based on various minimum desirable differences to detect. These numbers are per treatment arm and have 1-sided alphas of 0.05. It can be seen that a 9% difference would be detectable in a trial with 444 men in each group (90% power) and a 6% difference with 721 men in each group (80% power). 721 men per arm would give approximately 90% power to detect differences of 7%.

Table 5

Number required per arm

Difference	80% power	90% power
**5%**	**1039**	**1439**
6%	721	**999**
**7%**	**530**	734
**8%**	**406**	**562**
**9%**	**321**	**444**
**10%**	**260**	**360**

It is also anticipated that there will be about a 6% difference between the experimental arms in ≥Grade 2 bowel toxicity. This is based on results from the MD Anderson Trial [10, 69] which showed a 14% increase (12% vs. 26%) for an 8Gy dose difference (70 vs. 78Gy) and the RMH dose escalation trial [9] which showed a 12% increase (11% vs. 23%) for a 10Gy dose difference (64 vs. 74Gy). Taken together these results suggest an approximate 1.5% increase in bowel side effects for each 1Gy increase in dose (coincidentally similar to the $\gamma(50)$ for PSA response - see below). If the standard (2Gy) group has a ≥2 RTOG toxicity level of 10% then using a 2-sided alpha of 0.05 we would require 492 patients per arm (80% power) and 659 patients per arm for 90% power to exclude ≥16% toxicity rate in either experimental arm.

Concerning the radiobiology of prostate cancer a relatively conservative estimate of the slope of the dose/control curve, $\gamma(50)=1.5$ [69, 70], appropriate for the heterogeneous population of patients which may be recruited to CHHIP, has been used to calculate expected changes in PSA control. Table 6 shows predicted changes in PSA control compared to the standard 74Gy arm for various values of the α/β ratio. There would be equivalence between 74Gy and 60Gy if $\alpha/\beta=2.5$, and 74Gy and 57Gy if $\alpha/\beta=1.5$. The difference between the two experimental arms is expected to be approximately 6%. A difference of 6% would also be seen between the standard arm and the 60Gy arm if $\alpha/\beta=1.5$ or the standard arm and the 57Gy arm if $\alpha/\beta=2.5$. For α/β outside this range of 1.5 to 2.5 the difference in PSA control would be larger and within this range, smaller. Confidence limits on the estimates of the alpha beta ratio will be calculated using Bentzen's method [45].

Table 6 Predicted % change in PSA control rate ($\gamma(50)=1.5$)

**Dose**	**α/β ratio**						
	**1.0**	1.5	**2.0**	2.5	**3.0**	**5.0**	**10.0**
**60Gy**	**+9**	+6	**+1.5**	**0**	**-3**	**-7.5**	**-12**
**57Gy**	**+3**	**0**	**-4.5**	-6	**-9**	**-13.5**	**-18**

11 Research Governance

11.1.1 Trial Administration and Logistics

The Institute of Cancer Research (ICR) is the Sponsor of this study in line with the Research Governance Framework for Health and Social Care and the principles of GCP.

The Chief Investigator is Professor David Dearnaley. ICR-CTSU has overall responsibility for facilitating and coordinating the conduct of the trial and is also responsible for collating data obtained, and undertaking and reporting interim and final analyses.

11.1.2 Participating centres responsibilities

Centres wishing to recruit to this study will be asked to provide evidence that they can deliver protocol treatment. This will include the successful completion of the CHHIP QA programme.

Responsibilities are defined in an agreement between an individual participating centre and The Institute of Cancer Research.

11.1.3 Quality Assurance of Radiotherapy Delivery

The following QA steps and exercises must be completed by new centres in order to progress:

Questionnaires, planning exercises successfully completed and process document drafted before 1st randomisation; case reviews performed before treatment starts for 1st 2/3 patients; audit visit within 6 months of commencement of patient recruitment – dose point & film measurements.

11.2 Investigator training

Prior to commencing trial recruitment, training will be provided to identify key individuals in each participating network by the Chief Investigator and Trial Management Group. Training will include discussion on the background to the study, evidence for potential benefits and drawbacks of hypofractionation and discussion on the issues of clinical equipoise. Experience developed from successfully recruiting centres and information from associated studies will be provided to participants at their initial training and subsequently on a regular basis. Participating centres will be expected to join regular (≤3 monthly) teleconferences with the Trial Management Group to discuss trial progress and identify any recruitment, treatment planning and delivery difficulties and maintain standards determined by the Quality Assurance Group.

11.3 Case Report Forms

Case Report Forms (CRFs) which are in the form of a booklet should be completed for all patients and should not be made available to third parties.

CRFs should be completed as indicated in the Investigator's brochure. CRFs are in duplicate. The completed top copy must be sent by the hospital to ICR-CTSU as soon as they are due. The bottom copy must be retained in the booklet and held by the investigator. If information is not known it must be clearly stated.

The Trial Management Group reserves the right to amend or add to the CRFs as appropriate. Such changes do not constitute a protocol amendment, and revised or additional forms should be used by centres with immediate effect.

11.4 Protocol compliance/on site Monitoring

The CHHIP trial is being conducted in accordance with the professional and regulatory standards required for non-commercial research in the NHS under the Research Governance Framework for Health and Social Care and ICH GCP.

Participating centres may be monitored by ICR-CTSU and possibly by Health Authorities to carry out source data verification, and confirm compliance with the

protocol and the protection of patients' rights as detailed in the Declaration of Helsinki adopted by the 18th World Medical assembly, Helsinki, Finland, 1964 and later revisions (last revised Edinburgh 2000)[35]. Copies of the Declaration may be obtained from ICR-CTSU on request. By participating in the CHHIP trial the Principal Investigators at each centre are confirming agreement with his/her local NHS Trust to ensure that:

- Sufficient data is recorded for all participating patients to enable accurate linkage between hospital records and CRFs;
- Source data and all trial related documentation are accurate, complete, maintained and accessible for monitoring and audit visits;
- All staff at their centre who are involved with the trial are trained appropriately;
- All original Consent Forms are dated and signed by both the patient and investigator, and are kept together in a central log together with a copy of the specific patient information sheet(s) they were given at the time of consent.
- Copies of CRFs are retained for 15 years to comply with international regulatory requirements;
- Staff will comply with the Standard Operating Procedures for CHHIP trial.

ICR-CTSU will monitor receipt of CRFs. They will also check incoming CRFs for compliance with the protocol, inconsistent and missing data.

ICR-CTSU will contact centres to discuss dates of any proposed monitoring visits. Once a date has been confirmed a list of names of patients whose notes will be monitored during the visit will be sent to the centre. This list will be sent out in advance to give sufficient time for the notes to be made available. Site monitoring will usually be conducted at participating centres at least once during the first year following entry of the first patient. It is likely that a random sample of notes will be selected for limited source document verification.

11.5 Trial Management

11.5.1 Trial Management Group

A Trial Management Group (TMG) will be set up and will include the Chief Investigator (Professor David Dearnaley), co-investigators and identified collaborators, the trial statistician and the trial co-ordinators. Principal investigators and key study personnel will be invited to join the TMG as appropriate to ensure representation from a range of centres and professional groups. Not withstanding the legal obligations of the Sponsor and Chief Investigator, the TMG has operational responsibility for the conduct of the trial.

11.5.2 Trial Steering Committee

A Trial Steering Committee (TSC) will monitor and supervise the progress of the trial. The role of the TSC is to provide overall supervision of the trial on behalf of the funding body. In particular, the TSC will concentrate on the progress of the trial, adherence to the protocol, patient safety and the consideration of new information. Day-to day management of the trial is the responsibility of the Chief Investigator and TMG.

Membership will be limited and include an independent Chairman (not involved directly in the trial other than as a member of the TSC), not less than two other independent members, the Chief Investigator and the trial statistician.

Where possible membership will include a lay/consumer representative. Trial co-ordinators and other key members of the TMG will attend meetings (as observers) as appropriate. Observers from the funding body and, if applicable, host institutions or sponsors will be invited to all meetings. The TSC will meet at least annually.

11.5.3 Data Monitoring Committee

An independent Data Monitoring and Ethics Committee (DMEC) will be established to oversee the safety and interim efficacy of the trial. This committee will be constituted according to MRC Good Clinical Practice (MRC GCP). The DMEC will meet on a

regular basis as they see fit, but no less than annually. Following each meeting, the DMEC will report their findings and recommendations to the TSC and to the TMG.

11.6 End of Study

For the purposes of ethics approval, the study end date is deemed to be the date of the last data capture.

11.7 Archiving

Essential documents are documents that individually and collectively permit evaluation of the conduct of the trial and the quality of the data produced, for example CRFs, patient consent forms. These will be maintained at ICR-CTSU and at the Investigator Sites in a way that will facilitate the management of the trial, audit and inspection. They will be retained for a sufficient period (at least 15 years) for possible audit and inspection by the regulatory authority. The sponsor or trial organisers will notify the investigator sites of their responsibility for archiving essential documents. Documents will be securely stored and access will be restricted to authorised personnel. An archive log will be maintained to track archived documents

11.8 Publishing policy

All publications and presentations relating to the trial will be authorised by the TMG. A Writing Committee may be appointed. Authorship will be determined by the TMG and will include the Chief Investigator, co-investigators, and trial statisticians. Further authorship will be determined by centre accrual. All participating centres will be acknowledged in the manuscripts according to patient accrual.

12 Confidentiality and Liability

12.1 Liability/Indemnity/Insurance

This study is an investigator-led trial endorsed by the Clinical Trials Awards and Advisory Committee (CTAAC) of Cancer Research UK and the UK Medical Research

Council. Indemnity for participating hospitals is provided by the usual NHS indemnity arrangements.

12.2 Patient Confidentiality

The patient's full name, date of birth, hospital number and NHS number will be collected at randomisation to allow tracing through national records. The personal data recorded on all documents will be regarded as confidential, and to preserve each subject's anonymity, only their initials and date of birth will be recorded on subsequent Case Report Forms.

The investigator must keep a separate log of patients' trial numbers, names, and hospital numbers. The investigator must maintain in strict confidence trial documents, which are to be held in the local hospital (e.g. patients' written consent forms). The investigator must ensure the patient's confidentiality is maintained.

ICR-CTSU will maintain the confidentiality of all subject data and will not reproduce or disclose any information by which subjects could be identified, other than reporting of serious adverse events. Representatives of the trial team will be required to have access to patient notes for quality assurance purposes but patients should be reassured that their confidentiality will be respected at all times. In the case of special problems and/or competent authority queries, it is also necessary to have access to the complete study records, provided that patient confidentiality is protected.

13 Ethical Considerations

The trial will be performed in accordance with the recommendations guiding physicians in biomedical research involving human subjects adopted by the 18th World Medical assembly, Helsinki, Finland, 1964 and later revisions (last revised Edinburgh 2000) [73]

It is the responsibility of the Chief Investigator to obtain the required regulatory approval (Clinical Trial Authorisation) and a favourable ethical opinion (main REC approval).

It is the responsibility of the Principal Investigator at each participating Trust to obtain site-specific approval of the trial protocol and any subsequent amendments. All correspondence with the local REC should be filed by the Investigator.

It is the responsibility of the Investigator to give each patient, prior to inclusion in the trial, full and adequate verbal and written information regarding the objective and procedures of the trial and the possible risks involved. Patients must be informed about their right to withdraw from the trial and the possible risk involved. Written patient information must be given to each patient before enrolment. The written patient information is an approved patient information sheet according to national guidelines. This outlines the Quality of Life study (now closed to patient recruitment), and the collection of biological specimens. Patients will be encouraged to participate in these associated studies but if they decline, this will not exclude them from the main trial.

It is the responsibility of the Principal Investigator to obtain signed informed consent from all patients prior to inclusion in the trial.

14 Withdrawal of patients from study treatment

Patients who do not receive their allocated treatment for any reason should be treated at the discretion of their clinician. However, analyses of all outcome data will be on the basis of intention to treat. Unless the patient requests otherwise, all CRFs, including long term follow up, should be completed, regardless of treatment actually received. A trial deviation form should be completed to record details of deviation from treatment allocation, and also for any patient who withdraws consent for further follow up. Patients are asked prior to randomisation to consent to follow up should they withdraw from the treatment allocation (see patient information sheet and consent form), and any patient unwilling to give that assurance prior to trial entry should not be randomised. Patients are; however, free to reverse that decision at any time without giving a reason.

15 Financial Matters

The trial is investigator designed and led, and has been approved by the Clinical Trials Awards and Advisory Committee (CTAAC). It is endorsed by Cancer Research UK and meets the criteria for R&D support as outlined in the Statement of Partnership on Non-Commercial R&D in the NHS in England.

Research costs (to ICR-CTSU) are being funded by Cancer Research UK. If additional financial support is received from any other source, this will be made apparent to the approving Main REC and CTAAC, but will not require a protocol amendment.

No individual per patient payment will be made to trusts or investigators, but NCRN (or regional equivalent) network resources should be made available as the trial is part of the NCRI portfolio by virtue of its approval by CTAAC.

16 Associated studies

16.1 Quality of Life (QOL)

This sub-study has now closed to recruitment.

Quality of Life is an important secondary endpoint of the trial and forms an integral part of the protocol. Patients will be informed in the patient information sheet that they will have their QOL assessed regularly while involved in the CHHiP trial.

Patients who are entered into the CHHiP Trial and are willing and able to complete the self-report QOL questionnaires are eligible to enter the QOL sub-study and will be asked to give written informed consent for their participation.

Questionnaire(s) will be given in clinic at the following times, when the patient visits for their clinic appointment.

- Initial Assessment
- Pre- Radiotherapy
- Week 10

- Post Radiotherapy (6 months)

To avoid bias, the questionnaires should be completed by the patient before they see their clinician.

The next questionnaire(s) will be posted to the patient at their home address from time to time by the Clinical Trials & Statistics Unit at the Institute of Cancer Research, and would like them completed as follows:

- at 1 year
- at 18 months
- at 2 year and then annually to 5 years.

Before the questionnaires are sent, the patient's GP or the centre will be contacted to confirm that they are fit and well to receive the questionnaire. In all cases, the Clinical Trials & Statistics Unit will send a stamped addressed envelope to the patient to return the questionnaire. If the patients agree to participate in the Quality of Life study they will need to complete a demographics form.

Patients will be asked to fill out the questionnaires themselves as completely and accurately as possible. The average time to complete the questionnaire is 10-15 minutes.

The instruments used will be the FACT-P (Prostate) and UCLA/RAND Prostate Cancer Index. However, the UCLA/RAND Prostate Cancer Index will be replaced with the updated version. The other instruments to be used in new patients only will be the combined versions of the EPIC plus SF-12 questionnaires.

Patients already randomised to the study will receive the CHHiP questionnaire booklet version 2.1 (which includes FACT-P (Prostate), the only change will be that they will receive the updated version of the UCLA/RAND Prostate Cancer Index.

All newly randomised patients following approval of amendment no.5 will receive the CHHiP questionnaire booklet version 3.0 that will include the updated version of the

UCLA/RAND Prostate Cancer Index, EPIC (- hormone section) and the SF-12 questionnaires.

16.2 Health Economics

Health economic data will be collected via patient and clinician completed health resource usage questionnaires administered at 6, 12, 24, 36, 48 and 60 months. Analysis will take place once data on the principal clinical endpoints has been analysed and published. The data will be collated in a similar way to MRC RT01 and analyses of that trial will inform evaluation.

16.3 Translational studies

Four translational projects will be progressed.

(i) Tissue microarray analysis of diagnostic biopsies and prognosis. Patients' original needle guided biopsies of the prostate will be collected and prepared for tissue microarray analysis using new methodologies developed at the Institute of Cancer Research for these samples. Assessment will include prognostic factors for the development of progressive disease and, more specifically, for response to radiotherapy - for example, hypoxia and proliferative markers.

(ii) Collection of germline DNA for contribution to UK RAPPER and EU GENEPI studies.

These projects explore the relationship between germline polymorphisms and the development of radiation related normal tissue side effects. Blood samples will be collected for inclusion in these programmes.

(iii) Collection of dose cube information for the modelling of normal tissue complication effects using conventional and, uniquely, the hypofractionated radiotherapy schedules. Dose cube data will be collected using software developed at the Institute of Cancer Research. Detailed dose volume and

surface histogram analysis will be made and correlated with the development of radiation side effects and quality of life questionnaires.

(iv) To link the databases in (ii) and (iii) which will give a unique opportunity to explore detailed physical and biological parameters which may predict the development of radiation sequelae.

17. References

1. Doneux, A., et al., *The utility of digital rectal examination after radical radiotherapy for prostate cancer.* Clin Oncol (R Coll Radiol), 2005. **17**(3): p. 172-3.
2. *Consensus conference. The management of clinically localized prostate cancer.* JAMA, 1987. **258**(19): p. 2727-30.
3. *COIN Guidelines for the Management of Prostate Cancer.* Clin Oncol (R Coll Radiol), 1999. **11**(4): p. 135-72.
4. Fuks, Z. and A. Horwich, *Clinical and technical aspects of conformal therapy.* Radiotherapy and Oncology, 1993. **29**(2): p. 219-220.
5. Hanks, G.E., et al., *Dose escalation with 3D conformal treatment: five year outcomes, treatment optimization, and future directions.* Int J Radiat Oncol Biol Phys, 1998. **41**(3): p. 501-10.
6. Sandler, H.M., et al., *Dose escalation for stage C (T3) prostate cancer: minimal rectal toxicity observed using conformal therapy.* Radiotherapy and Oncology, 1992. **23**(1): p. 53-54.
7. Zelefsky, M.J., et al., *Dose escalation with three-dimensional conformal radiation therapy affects the outcome in prostate cancer.* Int J Radiat Oncol Biol Phys, 1998. **41**(3): p. 491-500.
8. Dearnaley, D.P., et al., *Comparison of radiation side-effects of conformal and conventional radiotherapy in prostate cancer: a randomised trial.* The Lancet, 1999. **353**: p. 267- 272.
9. Dearnaley, D.P., et al., *Phase III pilot study of dose escalation using conformal radiotherapy in prostate cancer: PSA control and side effects.* Br J Cancer, 2005. **92**(3): p. 488-98.
10. Pollack, A., et al., *Prostate cancer radiation dose response: results of the M. D. Anderson phase III randomized trial.* Int J Radiat Oncol Biol Phys, 2002. **53**(5): p. 1097-105.
11. *Zeitman, JAMA, 2005, 294 (10) 1233-9.*
12. Fiveash, J.B., et al., *3D conformal radiation therapy (3DCRT) for high grade prostate cancer: a multi-institutional review.* Int J Radiat Oncol Biol Phys, 2000. **47**(2): p. 335-42.
13. Kupelian, P.A., et al., *Higher than standard radiation doses (> or =72 Gy) with or without androgen deprivation in the treatment of localized prostate cancer.* Int J Radiat Oncol Biol Phys, 2000. **46**(3): p. 567-74.
14. Zelefsky, M.J., et al., *High dose radiation delivered by intensity modulated conformal radiotherapy improves the outcome of localized prostate cancer.* J Urol, 2001. **166**(3): p. 876-81.
15. Valicenti, R., et al., *Survival advantage from higher-dose radiation therapy for clinically localized prostate cancer treated on the Radiation Therapy Oncology Group trials.* J Clin Oncol, 2000. **18**(14): p. 2740-6.
16. Laverdiere, J., et al., *The efficacy and sequencing of a short course of androgen suppression on freedom from biochemical failure when administered with radiation therapy for T2-T3 prostate cancer.* J Urol, 2004. **171**(3): p. 1137-40.
17. Pilepich, M.V., et al., *Phase III radiation therapy oncology group (RTOG) trial 86-10 of androgen deprivation adjuvant to definitive radiotherapy in locally advanced carcinoma of the prostate.* Int J Radiat Oncol Biol Phys, 2001. **50**(5): p. 1243-52.
18. D'Amico, A.V., et al., *6-month androgen suppression plus radiation therapy vs radiation therapy alone for patients with clinically localized prostate cancer: a randomized controlled trial.* JAMA, 2004. **292**(7): p. 821-7.

19. Denham, J.W., et al., *Short-term androgen deprivation and radiotherapy for locally advanced prostate cancer: results from the Trans-Tasman Radiation Oncology Group 96.01 randomised controlled trial.* Lancet Oncol, 2005. **6**(11): p. 841-50.
20. Bolla, M., et al., *Long-term results with immediate androgen suppression and external irradiation in patients with locally advanced prostate cancer (an EORTC study): a phase III randomised trial.* Lancet, 2002. **360**(9327): p. 103-6.
21. Granfors, T., et al., *Combined orchiectomy and external radiotherapy versus radiotherapy alone for nonmetastatic prostate cancer with or without pelvic lymph node involvement: a prospective randomized study.* J-Urol, 1998. **159**(6): p. 2030-4 issn: 0022-5347.
22. Pilepich, M.V., et al., *Androgen suppression adjuvant to radiotherapy in carcinoma of the prostate. long-term results of phase III RTOG study 85-31.* Int J Radiat Oncol Biol Phys, 2003. **57**(2 Suppl): p. S172-3.
23. Hanks, G.E., et al., *Phase III trial of long-term adjuvant androgen deprivation after neoadjuvant hormonal cytoreduction and radiotherapy in locally advanced carcinoma of the prostate: the Radiation Therapy Oncology Group Protocol 92-02.* J Clin Oncol, 2003. **21**(21): p. 3972-8.
24. Horwitz, E.M., et al., *Subset analysis of RTOG 85-31 and 86-10 indicates an advantage for long-term vs. short-term adjuvant hormones for patients with locally advanced nonmetastatic prostate cancer treated with radiation therapy.* Int J Radiat Oncol Biol Phys, 2001. **49**(4): p. 947-56.
25. Dearnaley, D.P., *Combined modality treatment with radiotherapy and hormonal treatment in localized prostate cancer*, in *New Perspectives in Prostate Cancer*, A. Belldegrun, R.S. Kirby, and D.W.W. Newling, Editors. 2000, Isis Medical Media Ltd: Oxford. p. 169-180.
26. Forman, J.D., et al., *Neoadjuvant hormonal downsizing of localized carcinoma of the prostate: effects on the volume of normal tissue irradiation.* Cancer Invest, 1995. **13**(1): p. 8-15.
27. Zelefsky, M.J., et al., *Neoadjuvant hormonal therapy improves the therapeutic ratio in patients with bulky prostatic cancer treated with three-dimensional conformal radiation therapy.* International Journal of Radiation Oncology, Biology, Physics, 1994. **755-761**.
28. D'Souza, W.D. and H.D. Thames, *Is the alpha/beta ratio for prostate cancer low?* Int J Radiat Oncol Biol Phys, 2001. **51**(1): p. 1-3.
29. Fowler, J., R. Chappell, and M. Ritter, *Is alpha/beta for prostate tumors really low?* Int J Radiat Oncol Biol Phys, 2001. **50**(4): p. 1021-31.
30. Fowler, J.F., R.J. Chappell, and M.A. Ritter, *The prospects for new treatments for prostate cancer.* Int J Radiat Oncol Biol Phys, 2002. **52**(1): p. 3-5.
31. King, C.R. and J.F. Fowler, *A simple analytic derivation suggests that prostate cancer alpha/beta ratio is low.* Int J Radiat Oncol Biol Phys, 2001. **51**(1): p. 213-4.
32. Brenner, D.J., *Toward optimal external-beam fractionation for prostate cancer.* Int J Radiat Oncol Biol Phys, 2000. **48**(2): p. 315-6.
33. Duchesne, G.M. and L.J. Peters, *What is the alpha/beta ratio for prostate cancer? Rationale for hypofractionated high-dose-rate brachytherapy.* Int J Radiat Oncol Biol Phys, 1999. **44**(4): p. 747-8.
34. Brenner, D.J., et al., *Direct evidence that prostate tumors show high sensitivity to fractionation (low alpha/beta ratio), similar to late-responding normal tissue.* Int J Radiat Oncol Biol Phys, 2002. **52**(1): p. 6-13.
35. Nahum, A.E., et al., *Incorporating clinical measurements of hypoxia into tumor local control modeling of prostate cancer: implications for the alpha/beta ratio.* Int J Radiat Oncol Biol Phys, 2003. **57**(2): p. 391-401.

36. Brenner, D.J., *Hypofractionation for prostate cancer radiotherapy--what are the issues?* Int J Radiat Oncol Biol Phys, 2003. **57**(4): p. 912-4.
37. Thames, H.D., Jr., et al., *Changes in early and late radiation responses with altered dose fractionation: implications for dose-survival relationships.* Int J Radiat Oncol Biol Phys, 1982. **8**(2): p. 219-26.
38. Brenner, D.J. and E.J. Hall, *Fractionation and protraction for radiotherapy of prostate carcinoma.* Int J Radiat Oncol Biol Phys, 1999. **43**(5): p. 1095-101.
39. Duncan, W., et al., *Carcinoma of the prostate: results of radical radiotherapy (1970-1985).* International Journal of Radiation Oncology, Biology, Physics, 1993. **26**(2): p. 203-210.
40. Read, G. and R.C.S. Pointon, *Retrospective study of radiotherapy in early carcinoma of the prostate.* British Journal of Urology, 1989. **63**(2): p. 191-195.
41. Collins, C.D., R.W. Lloyd-Davies, and A.V. Swan, *Radical external beam radiotherapy for localised carcinoma of the prostate using a hypofractionation technique.* Clinical Oncology, 1991. **3**(3): p. 127-132.
42. Lukka, H., et al., *A randomized trial comparing two fractionation schedules for patients with localized prostate cancer.* Int J Radiat Oncol Biol Phys, 2003. **57**(2 Suppl): p. S126.
43. Yeoh, E.E., et al., *Evidence for efficacy without increased toxicity of hypofractionated radiotherapy for prostate carcinoma: early results of a Phase III randomized trial.* Int J Radiat Oncol Biol Phys, 2003. **55**(4): p. 943-55.
44. Livsey, J.E., et al., *Hypofractionated conformal radiotherapy in carcinoma of the prostate: five-year outcome analysis.* Int J Radiat Oncol Biol Phys, 2003. **57**(5): p. 1254-9.
45. Bentzen, S.M. and M.A. Ritter, *The alpha/beta ratio for prostate cancer: what is it, really?* Radiother Oncol, 2005. **76**(1): p. 1-3.
46. Kupelian, P.A., et al., *Preliminary observations on biochemical relapse-free survival rates after short-course intensity-modulated radiotherapy (70 Gy at 2.5 Gy/fraction) for localized prostate cancer.* Int J Radiat Oncol Biol Phys, 2002. **53**(4): p. 904-12.
47. Mott, J.H., J.E. Livsey, and J.P. Logue, *Development of a simultaneous boost IMRT class solution for a hypofractionated prostate cancer protocol.* Br J Radiol, 2004. **77**(917): p. 377-86.
48. Akimoto, T., et al., *Rectal bleeding after hypofractionated radiotherapy for prostate cancer: correlation between clinical and dosimetric parameters and the incidence of grade 2 or worse rectal bleeding.* Int J Radiat Oncol Biol Phys, 2004. **60**(4): p. 1033-9.
49. Thames, H.D., et al., *Time-dose factors in radiotherapy: a review of the human data.* Radiother Oncol, 1990. **19**(3): p. 219-35.
50. Brenner, D.J., *Fractionation and late rectal toxicity.* Int J Radiat Oncol Biol Phys, 2004. **60**(4): p. 1013-5.
51. Sydes, M.R., et al., *Implementing the UK Medical Research Council (MRC) RT01 trial (ISRCTN 47772397): methods and practicalities of a randomised controlled trial of conformal radiotherapy in men with localised prostate cancer.* Radiother Oncol, 2004. 72(2): p. 199-211.
52. Diaz, A., et al., *Indications for and the significance of seminal vesicle irradiation during 3D conformal radiotherapy for localized prostate cancer.* International Journal of Radiation Oncology, Biology, Physics, 1994. **30**(2): p. 323-329.
53. Parker, C.C. and D.P. Dearnaley, *The management of PSA failure after radical radiotherapy for localised prostate cancer.* Radiotherapy and Oncology, 1998. **49**(2): p. 103-110.
54. Padhani, A.R., et al., *Evaluating the effect of rectal distension and rectal movement on prostate gland position using cine MRI.* Int. J. Radiation Biol. Phys., 1999. **44**(3): p. 525-533.

55. Fiorino, C., et al., *Relationships between DVHs and late rectal bleeding after radiotherapy for prostate cancer: analysis of a large group of patients pooled from three institutions.* Radiother Oncol, 2002. **64**(1): p. 1-12.
56. Fiorino, C., et al., *Rectal dose-volume constraints in high-dose radiotherapy of localized prostate cancer.* Int J Radiat Oncol Biol Phys, 2003. **57**(4): p. 953-62.
57. Lu, Y., et al., *A method of analyzing rectal surface area irradiated and rectal complications in prostate conformal radiotherapy.* Int J Radiat Oncol Biol Phys, 1995. **33**(5): p. 1121-5.
58. Storey, M.R., et al., *Complications from radiotherapy dose escalation in prostate cancer: preliminary results of a randomized trial.* Int J Radiat Oncol Biol Phys, 2000. **48**(3): p. 635-42.
59. Benk, V.A., et al., *Late rectal bleeding following combined x-ray and proton high dose irradiation for patients with stage T3-T4 prostate carcinoma.* International Journal of Radiation Oncology, Biology, Physics, 1993. **26**(3): p. 551-557.
60. Boersma, L.J., et al., *Estimation of the incidence of late bladder and rectum complications after high-dose (70-78 GY) conformal radiotherapy for prostate cancer, using dose-volume histograms.* Int-J-Radiat-Oncol-Biol-Phys, 1998. **41**(1): p. 83-92 issn: 0360-3016.
61. Jackson, A., et al., *Late rectal bleeding after conformal radiotherapy of prostate cancer. II. Volume effects and dose-volume histograms.* Int J Radiat Oncol Biol Phys, 2001. **49**(3): p. 685-98.
62. Wachter, S., et al., *Rectal sequelae after conformal radiotherapy of prostate cancer: dose-volume histograms as predictive factors.* Radiother Oncol, 2001. **59**(1): p. 65-70.
63. Marks, L.B., et al., *The response of the urinary bladder, urethra, and ureter to radiation and chemotherapy.* Int J Radiat Oncol Biol Phys, 1995. **31**(5): p. 1257-80.
64. Emami, B., et al., *Tolerance of normal tissue to therapeutic irradiation.* International Journal of Radiation Oncology, Biology, Physics, 1991. **21**(1): p. 109-22.
65. *Mangar, S et al., Evaluating the relationship between erectile dysfunction and dose received by the penile bulb. Radiother Oncology, 2006 in Press*
66. *Roach et al., Penile bulb dose and impotence after three-dimensional conformal radiotherapy for prostate cancer on RTOG 9407. Int J Radiat Oncol Biol Phys 2004;60:1351-6.*
67. *IPSM Report, Physics Med Biol 35 (10) ppl 355-60*. 1990,.
68. A'Hern, R.P., *Sample size tables for exact single-stage phase II designs.* Stat Med, 2001. **20**(6): p. 859-66.
69. Cheung, R., et al., *Dose-response for biochemical control among high-risk prostate cancer patients after external beam radiotherapy.* Int J Radiat Oncol Biol Phys, 2003. **56**(5): p. 1234-40.
70. Levegrun, S., et al., *Risk group dependence of dose-response for biopsy outcome after three-dimensional conformal radiation therapy of prostate cancer.* Radiother Oncol, 2002. **63**(1): p. 11-26.
71. Pollack, A., et al., *Preliminary results of a randomized radiotherapy dose-escalation study comparing 70 Gy with 78 Gy for prostate cancer.* J Clin Oncol, 2000. **18**(23): p. 3904-11.
72. Peeters, S. T., W. D. Heemsbergen, et al. (2006). *"Dose-response in radiotherapy for localized prostate cancer: results of the Dutch multicenter randomized phase III trial comparing 68 Gy of radiotherapy with 78 Gy."* J Clin Oncol **24**(13): 1990-6.
73. http://www.wma.net/e/ethicsunit/helsinki.htm
74. http://www.nccn.org (10th November 2006)

75. Roach M, Hanks G, Thames H Jr et al. *Defining biochemical failure following radiotherapy with or without hormonal therapy in men with clinically localized prostate cancer: recommendations of the RTOG-ASTRO Phoenix Concensus Conference.* Int J Radiat Oncol Biol Phys 2006; **66**: 965-74
